# Trichloroacetic acid fueled practical amine purifications

**DOI:** 10.3762/bjoc.18.26

**Published:** 2022-02-24

**Authors:** Aleena Thomas, Baptiste Gasch, Enzo Olivieri, Adrien Quintard

**Affiliations:** 1 Aix-Marseille Univ, CNRS, Centrale Marseille, iSm2, Marseille, France

**Keywords:** amines, decarboxylation, eco-compatible, out of equilibrium, purification

## Abstract

Amine purification have for long been dominated by tedious stepwise processes involving the generation of large amounts of undesired waste. Inspired by recent work on out of equilibrium molecular machinery, using trichloroacetic acid (TCA), we disclose a purification technique considerably decreasing the number of operations and the waste generation required for such purifications. At first, TCA triggers the precipitation of the amines through their protonated salt formation, enabling the separation with the impurities. From these amine salts, simple decarboxylation of TCA liberates volatile CO_2_ and chloroform affording directly the pure amines. Through this approach, a broad range of diversely substituted amines could be isolated with success.

## Introduction

**Scheme 1:**
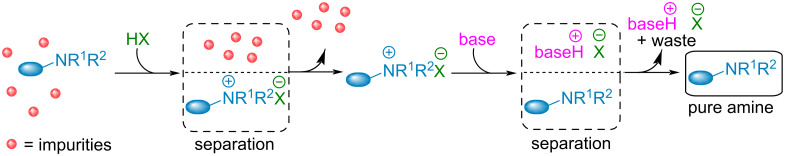
Classical amine purification.

Isolation of pure amines from reaction mixtures or natural extracts is crucial in modern organic chemistry. However, the most widely applied methods for these purifications have remained unchanged for decades. Aside from distillation or lengthy and costly chromatography techniques generating large amounts of waste, classical purification of amines found in practical textbooks [1–3] imply the temporary formation of a salt in the presence of an acid (Scheme 1). This strategy has been applied since the beginning of the 19th century as highlighted by the Sertürner isolation of morphine [4]. However, after formation of the amine acid (ammonium) salt and separation of the impurities, another separation is required to liberate again the free amine resulting in the undesired generation of waste. Most notably, this step can go through a liquid–liquid separation that requires multiple operations generating large amounts of waste notably arising from the different organic and aqueous layers.

In the context of the development of more eco-compatible organic synthesis limiting the number of operations and the associated generation of waste, discovery of alternative purification techniques is mandatory.

**Scheme 2:**
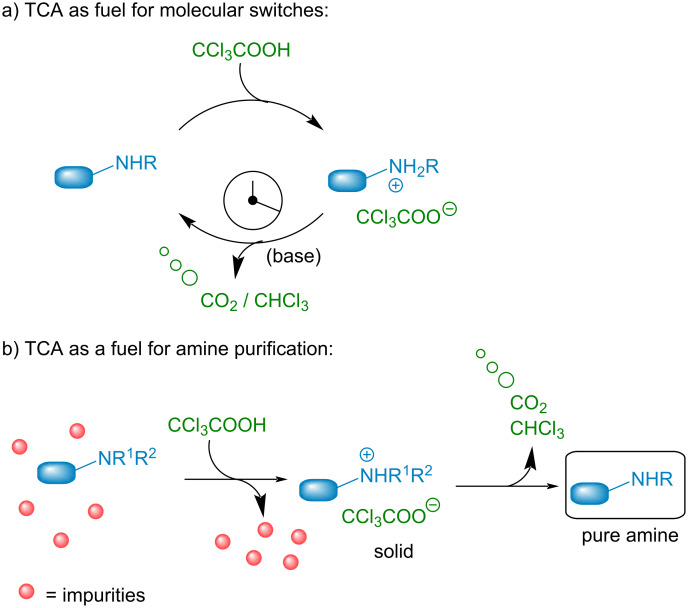
Principle of out-of equilibrium machinery using TCA (a) and our application to amines purification (b).

Avoiding waste accumulation, dissipative acids have recently found interesting applications in the field of out of equilibrium molecular switches and motors [5]. They enable the temporary formation of protonated amines which evolve spontaneously over time back again to the neutral amine upon release of CO_2_ from the initial acid. Among the potential acids available for such processes, trichloroacetic acid (TCA) has been used to fuel different supramolecular switches [6–10]. This cheap and simple acid enables a temporary protonation while time-controlled decarboxylation liberates volatile CO_2_ and chloroform as single waste (Scheme 2a). This strategy has been applied with success by the groups of Takata, Leigh, Kim and ours to time control different molecular switches ranging from rotaxanes, cucurbit[8]uril, to supramolecular smart materials [11–16]. TCA is also known for its application in the precipitation of proteins through what is believed to be an unfolding of the proteins structures [17–18].

In this context, we hypothesized that TCA could be used to facilitate amines purification considerably limiting waste generation and operations, which is to the best of our knowledge unprecedented (Scheme 2b). Addition of this acid to amines containing impurities could trigger the generation of a precipitating salt, enabling the separation from the impurities. From there, simple decarboxylation, spontaneous or catalyzed through the addition of a small amount of low boiling organic bases, would liberate the volatile wastes and the clean pure amine. Herein, we present our success in developing such a technique.

## Results and Discussion

In order to prove the feasibility of a TCA-induced amine purification, we first focused on the study of the different parameters crucial for such a process. For such purpose, we initially studied the purification of a model 1:1 mixture of dicyclohexylamine and naphthalene (Table 1). Gratifyingly, adding 3 equivalents of TCA to this mixture dissolved in EtOAc, precipitation of the amine salt enabled the removal of naphthalene in the solvent.

Beside the choice of the solvent for the crystallization which strongly depends on the solubility of the protonated amine salts involved, we first focused on the optimization of the decarboxylation conditions (Table 1, see Supporting Information File 1 for additional optimization). From the solid amine salt, different conditions were thus tested for a rapid decarboxylation. When stirring the amine salt in EtOAc, only a partial decarboxylation was observed even after 24 hours (Table 1, entry 1). Presence of TCA in the ^1^H NMR spectra is easily monitored through a broad peak around 9 ppm and also through the upfield migration of the other peaks of the amine ammonium salt. Heating the amine salt precipitate neat at 100 °C also provided partial decarboxylation (Table 1, entry 2). In sharp contrast, use of Lewis basic solvents such as DMF considerably accelerate the decarboxylation kinetic (Table 1, entry 3) with the recovery of the free amine after 24 hours. However, this solvent is not convenient given its high boiling point making difficult it’s separation from the purified amine. Accordingly, use of acetonitrile (Table 1, entry 4), also provides the free amine easily. Moreover, the solvent is easily evaporated concomitantly with the removal of the generated CO_2_ and chloroform, providing the isolated pure amine in 86% purification yield. This system is closely related to out of equilibrium supramolecular machinery through a temporary protonation returning to the initial state upon time [3–5]. While conceptually interesting, in terms of laboratory practicability, it would be desirable to have a faster release of the free amine. For this purpose, a small amount of volatile base (Et_3_N) was incorporated to catalyze the TCA decarboxylation while heating at 60 °C in a rotary evaporator under reduced pressure. Through this technique, the pure free amine could be isolated after 15 minutes of gentle heating, simultaneously evaporating the generated chloroform as well as CH_3_CN and Et_3_N (Table 1, entry 5). Overall, the TCA-induced purification protocol enables the convenient purification of the initial mixture with the isolation of pure dicyclohexylamine in an 94% yield. The excellent yield observed for the purification technique of this, high boiling amine, demonstrates all the potential of this system.

**Table 1:** Optimization of the decarboxylation.

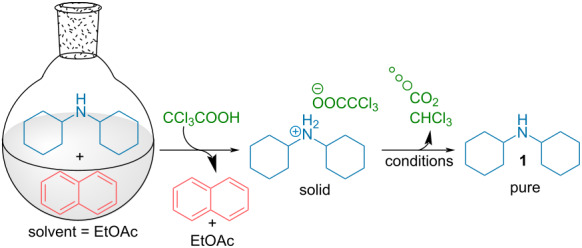		

Entry	Decarboxylation conditions	Result

1	EtOAc, 60°C, 24 h	partial TCA decarboxylation
2	neat, 100 °C, 1 h	partial TCA decarboxylation
3	DMF, 60°C, 15 min	complete TCA decarboxylation but difficulty in removing DMF
**4**	**CH** **_3_** **CN, rt, 24 h**	**complete TCA decarboxylation (86% purification yield)**
**5**	**CH** **_3_** **CN, Et** **_3_** **N (1.5 equiv), 60 °C, 15 min, vacuum** ^a^	**complete TCA decarboxylation (94% purification yield)**

^a^15 minutes heating performed in a rotary evaporator with a gradual decrease of the pressure from 100 to 10 mbar (see Supporting Information File 1).

In order to highlight the interest of this technique and its broad applicability, dicyclohexylamine could be purified from various mixtures containing 1 equivalent of structurally different impurities (Table 2). Aside from naphthalene (Table 2, entry 1), dicyclohexylamine could be isolated in its pure form starting from model mixtures containing various aromatics, phenols, alkanes or alkenes in overall purification yields ranging from 53 to 98%. The lower yields are observed for coordinating phenol and catechol (Table 2, entries 3 and 4), probably impacting the crystallization process, while less coordinating species all provided purification yields above 80%. Finally, polar DMF was removed from dicyclohexylamine in 81% yield (Table 2, entry 8).

**Table 2:** Examples of impurities removed from dicyclohexylamine.

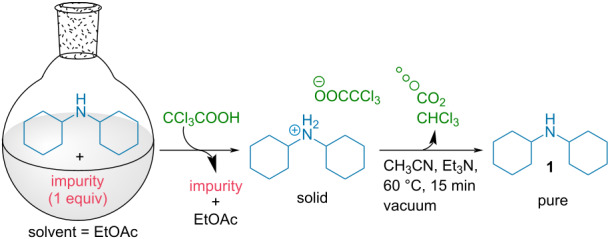		

Entry	Impurity (1 equiv)	Purification yield

1	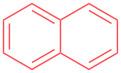	94%
2	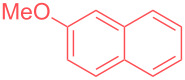	95%
3	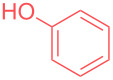	53%
4	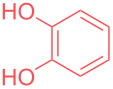	69%^a^
5	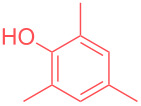	80%
6	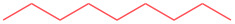	90%
7	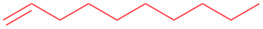	98%
8	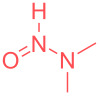	81%

^a^Contains 2% of catechol.

Aside from the potential impurities, we also focused on the purification of other types of amines (Table 3). One equivalent of naphthalene or 2-methoxynaphthalene were chosen as representative model impurities given their potential π-stacking ability and solid state at room temperature. Consequently, they are more challenging to separate from the generated amine salts, demonstrating the power of the method. Aside from secondary dicyclohexylamine (Table 3, entry 1), a broad range of primary amines could also be separated from the aromatic impurities (Table 3, entries 2–10). As for other purification techniques, the challenge lies in the identification of the appropriate solvent to dissolve well the initial mixture and induce the amine–TCA complex precipitation. For such purpose, EtOAc, pentane, CH_3_CN or Et_2_O have been used in this table depending on the solubility observed (see Supporting Information File 1 for details). Depending on the TCA–amine salt solubility in the solvent used, the purified primary amines could be isolated in 40–94% yield. Bulky 2,2,6,6-tetramethyl-4-piperidone could also be isolated from naphthalene in 94% yield through this approach (Table 3, entry 11). Other heterocyclic amines such as acridine or 1,2-dimethylimidazole could also be isolated with success in 53–65% yields (Table 3, entries 12 and 13). Of importance, this technique is also efficient for the isolation of highly complex natural amines such as brucin, isolated in 57% yield using TCA (Table 3, entry 14).

**Table 3:** Scope of purified amines.

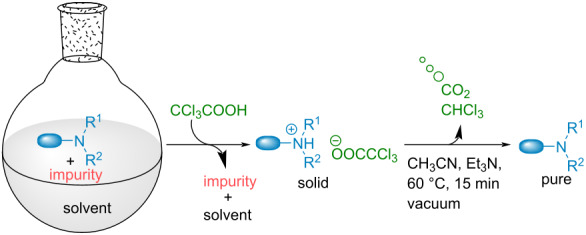			

Entry^a^	Amine/impurity (1:1 ratio)	Precipitation solvent	Amine purification yield

1	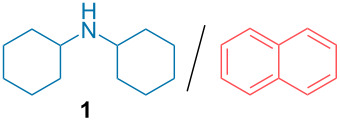	EtOAc	94%
2	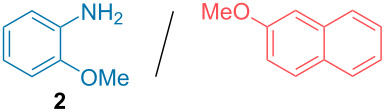	EtOAc	72%
3	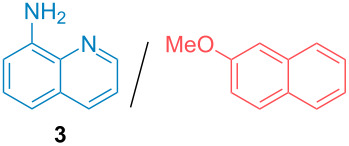	EtOAc	40%
4	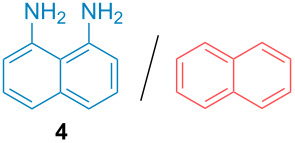	EtOAc	40%
5	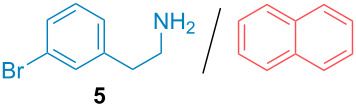	EtOAc	71%
6	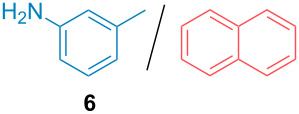	EtOAc	79%
7	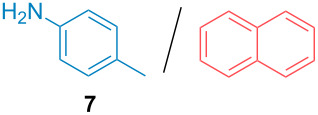	pentane	45%
8	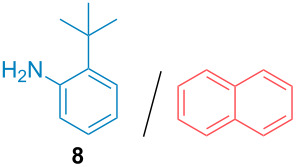	pentane	81%
9	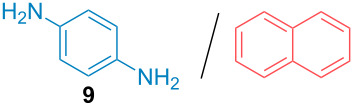	EtOAc	91%
10	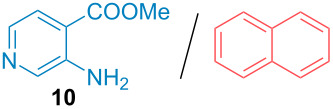	EtOAc	72%
11	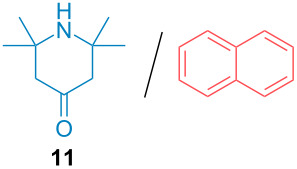	EtOAc	94%
12	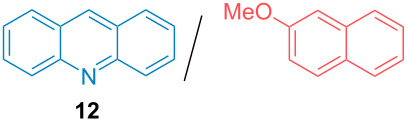	pentane-Et_2_O	65%
13	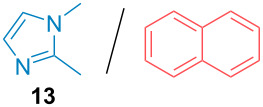	pentane	53%
14	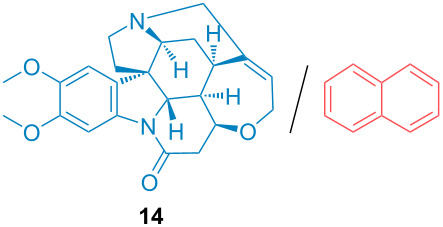	CH_3_CN	57%

^a^Depending on the amine, EtOAc, pentane, CH_3_CN or Et_2_O have been used as solvent (see Supporting Information File 1 for details).

As mentioned, identification of a suitable solvent solubilizing the initial mixture and enabling precipitation/crystallization of the amine salt is the key for success in this method. As a consequence, for now, we also experienced absence of amine salt precipitation using different other amines (see Supporting Information File 1 for details). However, we are convinced that simple solvent screening could unlock the purification of these other amines also enabling an increase in the purification yields.

**Scheme 3:**
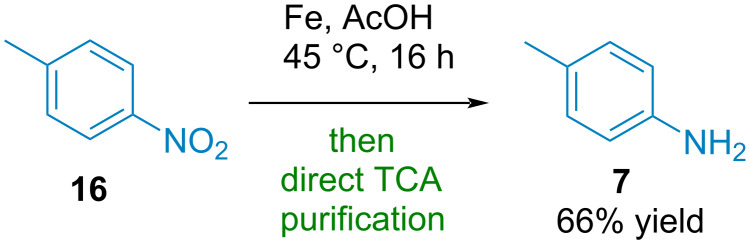
Application of the TCA purification from a crude reaction mixture.

Finally, in order to demonstrate the potential of the purification technique, we performed the reduction of 4-nitrotoluene and directly purified the crude mixture through the TCA purification protocol to obtain pure *p*-toluidine in 66% isolated yield (Scheme 3). This highlights the potential of this technique for organic synthesis.

## Conclusion

To conclude, we have disclosed a new approach considerably limiting waste and operations necessary for amine purification. Taking advantage of a temporary protonation with TCA, the solid amine salts generated can be separated from the impurities. Closely related to out of equilibrium systems applied in molecular switches and motors under temporal control, from the amine salt, isolation of the pure free amine occurs through a simple decarboxylation of TCA, releasing volatile CO_2_ and CHCl_3_. As a result, purification of these amines can proceed easily and with excellent yields (up to 98% overall purification yield). This method can virtually be applied for any amine purification given that an appropriate solvent is identified for the precipitation of the TCA–amine salt. Given the decrease in required operations and waste generation, this methodology should rapidly find applications in organic chemistry laboratories but also possibly on industrial scale.

## Experimental

**Typical experimental procedure with dicyclohexylamine 1:** Dicyclohexylamine (37 mg, 0.21 mmol, 1 equiv) and 2-methoxynaphthalene (33 mg, 0.21 mmol, 1 equiv) were dissolved in EtOAc (1 mL). TCA (100 mg, 0.62 mmol, 3 equiv) was then added at room temperature. The white precipitate formed was filtered and washed with EtOAc (2 × 2 mL). CH**_3_**CN (2 mL) and Et**_3_**N (0.04 mL, 0.32 mmol, 1.5 equiv) were then added and the solvent was evaporated at 60 °C for 15 min (100 to 10 mbar). The purified amine (35 mg, 0.2 mmol, 95% yield) was obtained as a colorless liquid. **^1^**H NMR (400 MHz, CDCl**_3_**) δ 2.56–2.49 (m, 2H), 1.85–1.80 (m, 4H), 1.72–1.66 (m, 4H), 1.60–1.54 (m, 2H), 1.27–0.94 (m, 10H) ppm.

## Supporting Information

File 1 Experimental details.
