# Supplementary material for: Trichloroacetic acid fueled practical amine purifications
Source: Beilstein J Org Chem. 2022 Feb 24;18:225–31. doi: 10.3762/bjoc.18.26 (PMC8895030; doi:10.3762/bjoc.18.26)
Supplement: File 1 — Experimental details. [file Beilstein_J_Org_Chem-18-225-s001.pdf]

BEILSTEIN JOURNAL OF ORGANIC CHEMISTRY

Supporting Information

for

Trichloroacetic acid fueled practical amine purifications

Aleena Thomas, Baptiste Gasch, Enzo Olivieri and Adrien Quintard

Beilstein J. Org. Chem. **2022,** *18,* 225–231. doi:10.3762/bjoc.18.26

Experimental details

License and Terms: This is a supporting information file under the terms of the Creative Commons Attribution License (https://creativecommons.org/licenses/by/4.0). Please note that the reuse, redistribution and reproduction in particular requires that the author(s) and source are credited and that individual graphics may be subject to special legal provisions.
The license is subject to the *Beilstein Journal of Organic Chemistry* terms and conditions: (https://www.beilstein-journals.org/bjoc/terms)

Table of Contents

General information... S3

Optimization of the decarboxylation…………………………….............................. S3

TCA as dissipative acid for amine purifications.. S4

Examples of amines who did not generate a TCA salt under our first attempts…….... S35

General information

NMR spectra were recorded on a Bruker AC 300 (300 MHz) or a Bruker AC 400 (400 MHz) spectrometer in $CDCl_3$. Chemical shifts are given in ppm, using as internal standards the residual solvent signal (δ = 7.26). Data for ^1H NMR are reported as follows: chemical shift (multiplicity [s = singlet, d = doublet, t = triplet, q = quadruplet, m = multiplet, br = broad], coupling constants *J* in Hertz (Hz), integration).

All reagents used are commercially available and were used directly without further purification. Given the toxicity of the generated chloroform, all experiments should be performed under fume-hoods.

Optimization of the decarboxylation:

Dicyclohexylamine **1** (37 mg, 0.21 mmol, 1 equiv.) and naphthalene (27 mg, 0.21 mmol, 1 equiv.) were dissolved in EtOAc (0.5 mL). TCA (100 mg, 0.62 mmol, 3 equiv.) was then added at room temperature. The generated white precipitate was filtered and washed with EtOAc (2 x 1 mL). From this white precipitate, various conditions were tested for the optimization of the decarboxylation to obtain purified amine as shown in the table below.

Decarboxylation conditions:

No.	Conditions	Results (purification yield)
1	EtOAc, 60°C, 24 h	Partial TCA decarboxylation
2	DMF, 60°C, 15 min (10-100 mbar)	Complete TCA decarboxylation but difficulty at removing DMF
3	CH_3CN, rt, 24 h	Complete TCA decarboxylation (86%)
4	CH_3CN, 60 °C, 15 min (10-100 mbar)	Partial TCA decarboxylation
5	Neat, 60 °C, 30 min (10-100 mbar)	Partial TCA decarboxylation
6	Neat, 100 °C, 1 h	Partial TCA decarboxylation
7	CH_3CN, 10 mol% Et_3N, rt, 24 h	Complete TCA decarboxylation (93%)
8	CH_3CN, 1.5 equiv. Et_3N, rt, 24 h	Complete TCA decarboxylation (91%)
9	CH_3CN, 1.5 equiv. Et_3N, 60 °C, 15 min (10-100 mbar)	Complete TCA decarboxylation (94%)

TCA as dissipative acid for amine purifications:

Dicyclohexylamine **1**

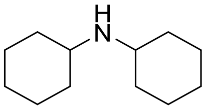

Dicyclohexylamine (37 mg, 0.21 mmol, 1 equiv.) and 2-methoxynaphthalene (33 mg, 0.21 mmol, 1 equiv.) were dissolved in EtOAc (1 mL). TCA (100 mg, 0.62 mmol, 3 equiv.) was then added at room temperature. The white precipitate formed was filtered and washed with EtOAc (2 x 2 mL). CH_3CN (2 mL) and Et_3N (0.04 mL, 0.32 mmol, 1.5 equiv.) were then added and the solvent was evaporated at 60 °C for 15 min (30-100 mbar). The purified amine (35 mg, 0.2 mmol, 94% yield) was obtained as a colorless liquid.

^{1}H NMR (400 MHz, $CDCl_3$): δ (ppm) 2.56-2.49 (m, 2H), 1.85-1.8 (m, 4H), 1.72-1.66 (m, 4H), 1.6-1.54 (m, 2H), 1.27-0.94 (m, 10H)

^{13}C NMR (75 MHz, $CDCl_3$): δ (ppm) 53.14, 34.39, 26.28, 25.40

Data in accordance with the literature.

Reference : https://sdbs.db.aist.go.jp/sdbs/cgi-bin/landingpage?sdbsno=2107

^{1}H NMR of the purified amine:

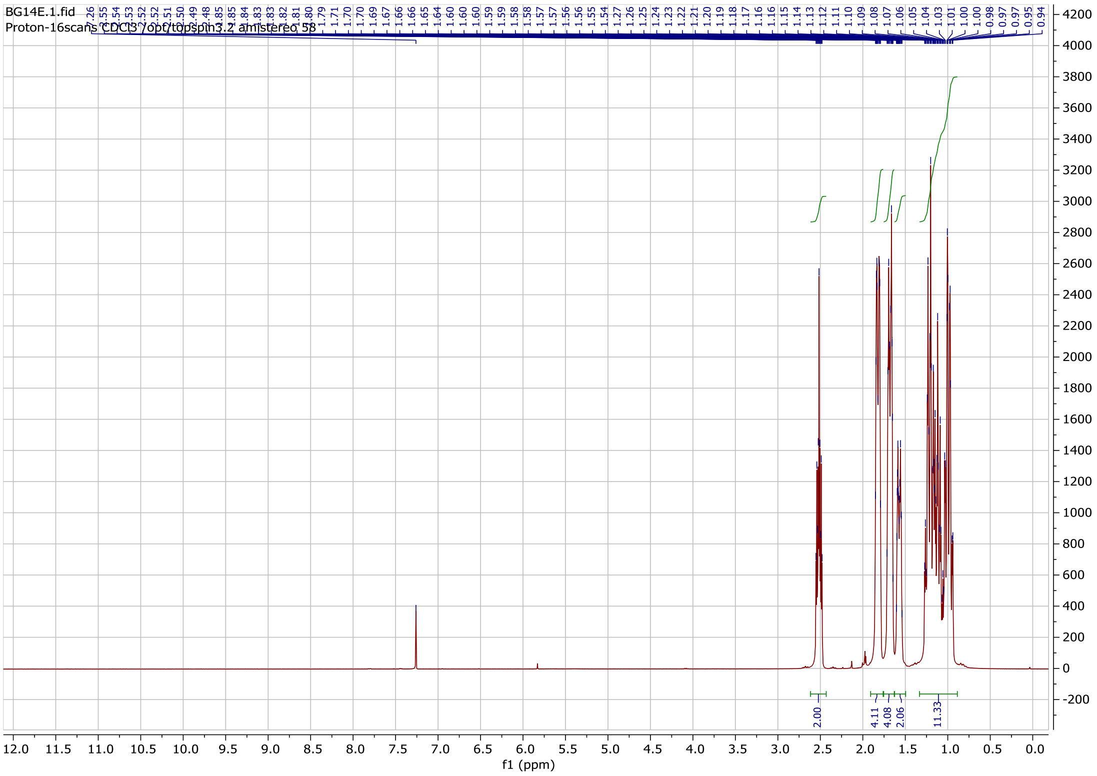

^{13}C NMR of the purified amine:

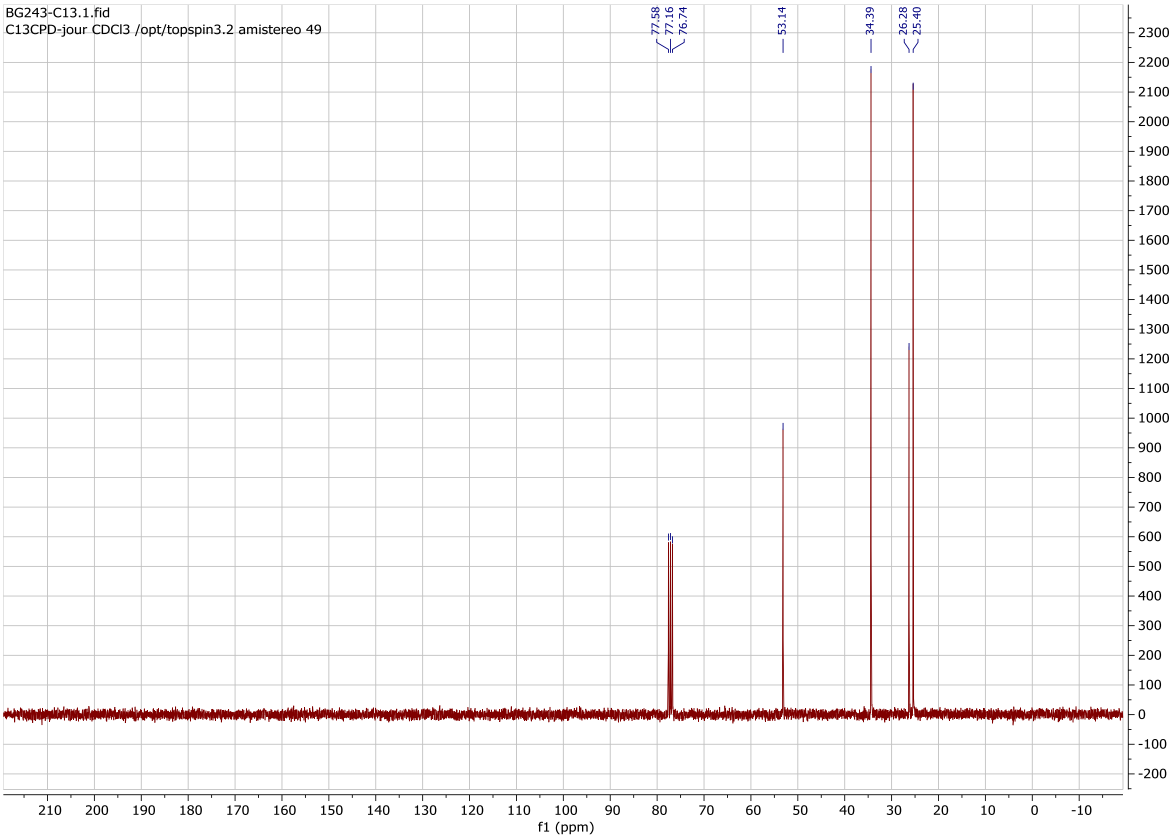

^{1}H NMR obtained with the decarboxylation condition of entry 1 (EtOAc alone): Presence of the TCA.amine salt

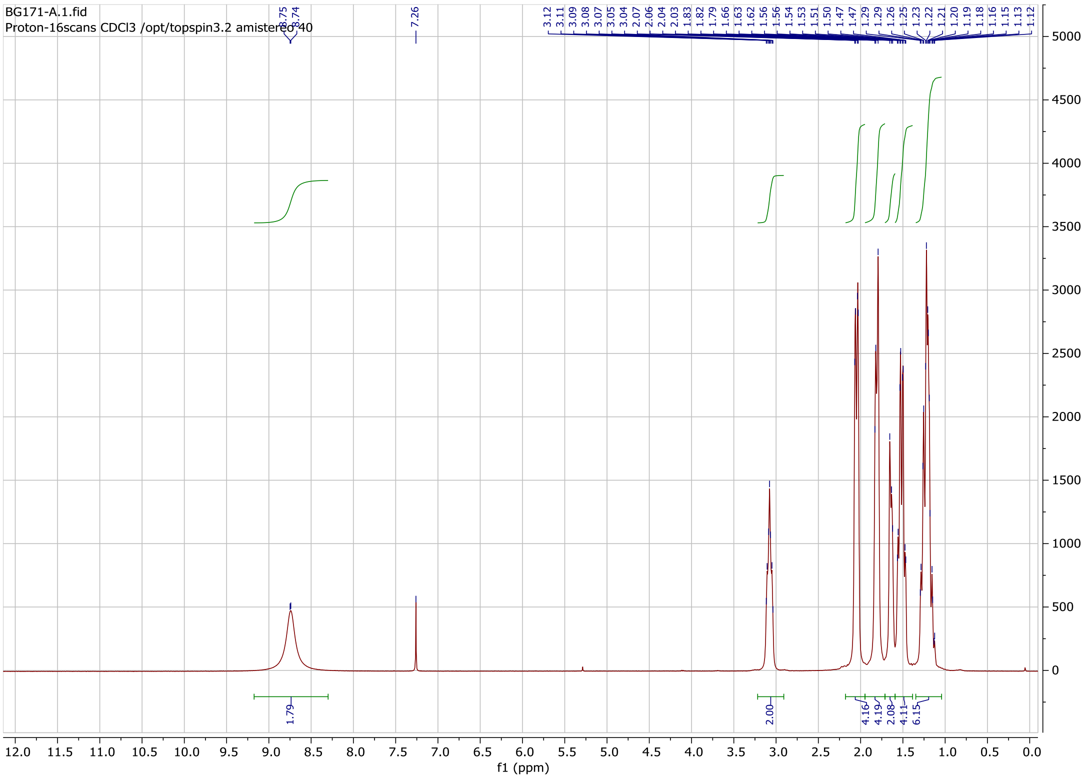

Various impurities tested with dicyclohexylamine following the same purification protocol:

No.	Impurity (0.21 mmol, 1 equiv.)	Results (purified amine)
1	Naphthalene	94%
2	2-methoxynaphthalene	95%
3	Phenol	53%
4	Mesitol	80%
5	Catechol	69%
6	Nonadecane	90%
7	1-decene	98%
8	DMF	81%

o-anisidine **2**

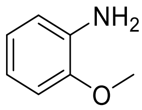

o-anisidine (26.8 mg, 0.21 mmol, 1 equiv.) and 2-methoxynaphthalene (33 mg, 0.21 mmol, 1 equiv.) were dissolved in EtOAc (1 mL). TCA (100 mg, 0.62 mmol, 3 equiv.) was then added at room temperature. The precipitate formed was filtered and washed with ice cold EtOAc (2 x 2 mL). CH_3CN (2 mL) and Et_3N (0.04 mL, 0.32 mmol, 1.5 equiv.) were added and the solvent was evaporated at 60 °C (30-100 mbar) for 15 min to obtain the purified amine (19.5 mg, 0.15 mmol, 72% yield).

^{1}H NMR (400 MHz, $CDCl_3$): δ (ppm) 6.82-6.71 (m, 4H), 3.86 (s, 3H)

^{13}C NMR (75 MHz, $CDCl_3$): δ (ppm) 147.44, 136.27, 121.18, 118.57, 115.13, 110.57, 55.52

Data in accordance with literature.

Reference : https://sdbs.db.aist.go.jp/sdbs/cgi-bin/landingpage?sdbsno=1475

^{1}H NMR of the purified amine:

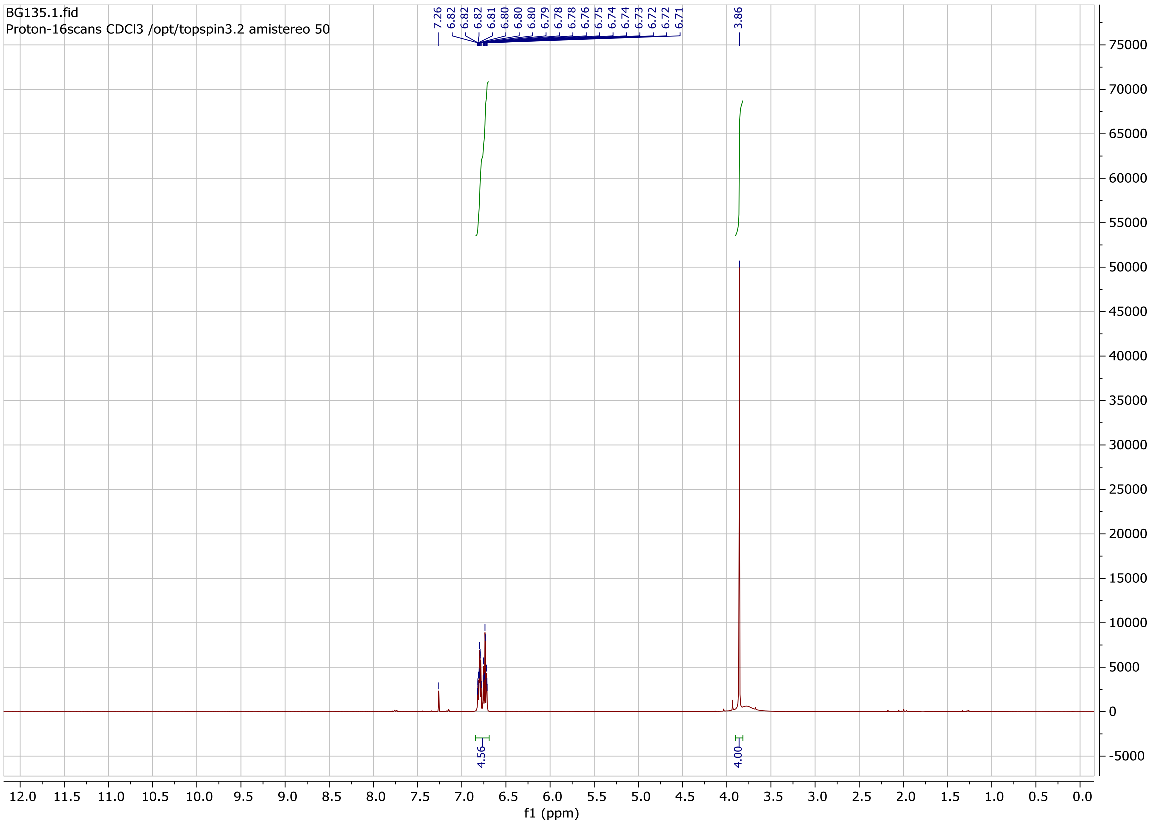

^{13}C NMR of the purified amine:

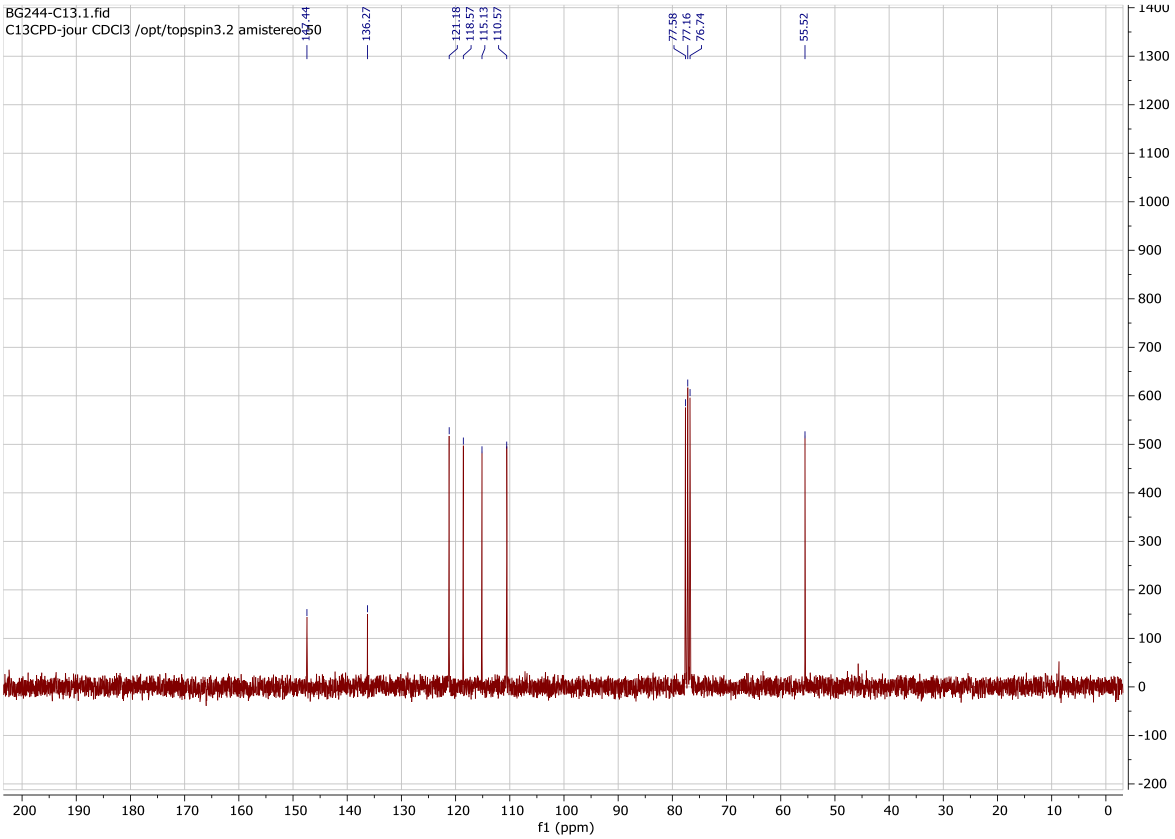

8-aminoquinoline **3**

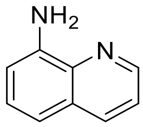

8-aminoquinoline (30 mg, 0.21 mmol, 1 equiv.) and 2-methoxynaphthalene (33 mg, 0.21 mmol, 1 equiv.) were dissolved in EtOAc (1 mL). TCA (100 mg, 0.62 mmol, 3 equiv.) was then added at room temperature. The precipitate formed was filtered and washed with EtOAc (2 x 2 mL). CH_3CN (2 mL) and Et_3N (0.04 mL, 0.32 mmol, 1.5 equiv.) were added and the solvent was evaporated at 60 °C (10-100 mbar) for 15 min to obtain the purified amine (12.4 mg, 0.08 mmol, 40% yield).

^{1}H NMR (400 MHz, $CDCl_3$): δ (ppm) 8.77 (dd, *J* = 1.6; 4.1 Hz, 1H), 8.06 (dd, *J* = 1.6; 8.4 Hz, 1H), 7.38-7.31 (m, 2H), 7.17-7.14 (m, 1H), 6.94-6.92 (m, 1H), 4.97 (br, 2H)

^{13}C NMR (75 MHz, $CDCl_3$): δ (ppm) 147.53, 144.08, 138.54, 136.08, 128.96, 127.48, 116.13, 110.14

Data in accordance with the literature.

Reference :https://www.sigmaaldrich.com/deepweb/assets/sigmaaldrich/quality/spectra/308/009/FNMR004723.pdf

^{1}H NMR of the purified amine:

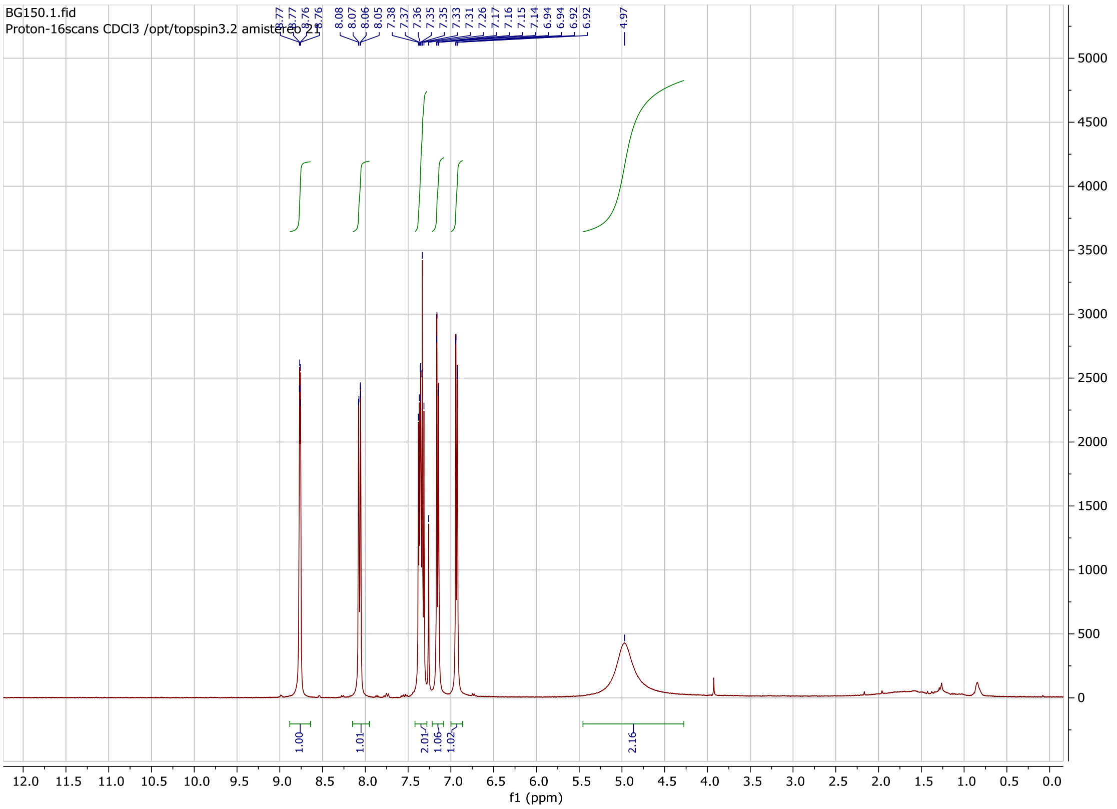

^{13}C NMR of the purified amine:

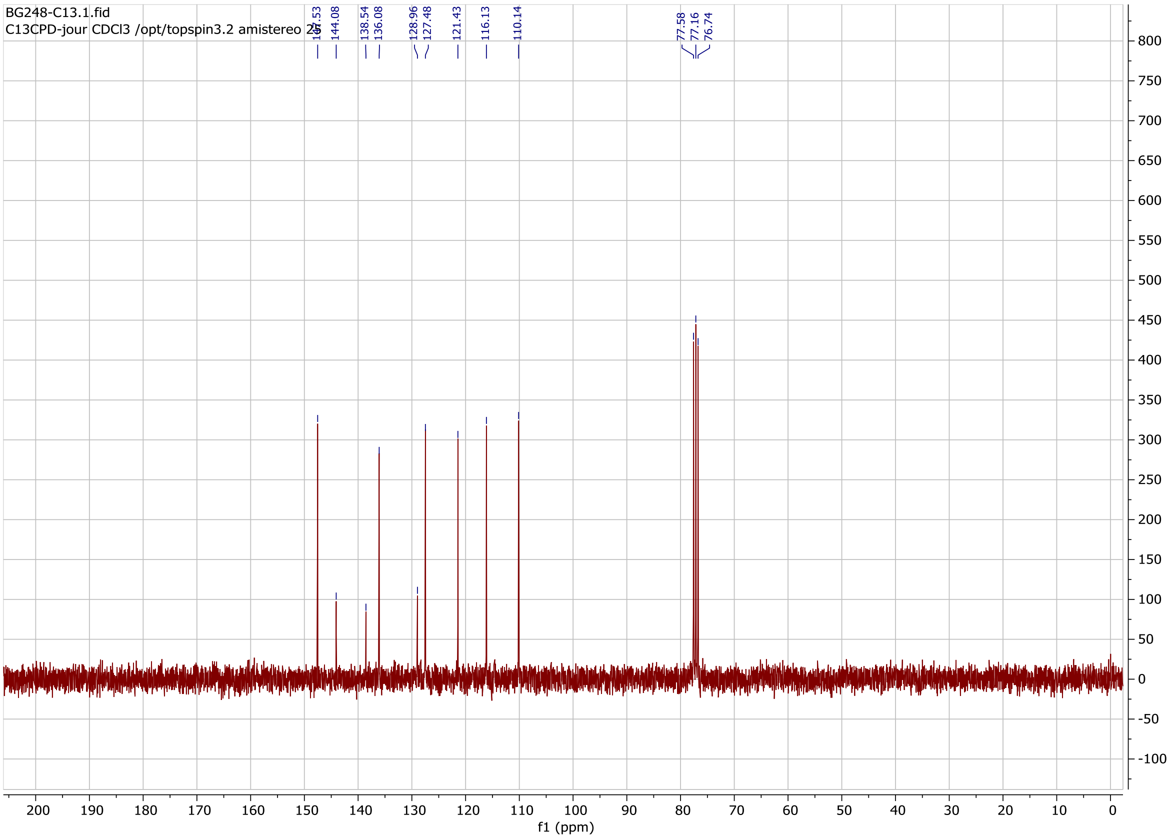

1,8-diaminonaphthalene **4**

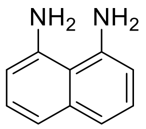

1,8-diaminonaphthalene (31 mg, 0.21 mmol, 1 equiv.) and naphthalene (27 mg, 0.21 mmol, 1 equiv.) were dissolved in EtOAc (1 mL). TCA (100 mg, 0.62 mmol, 3 equiv.) was added at room temperature. The precipitate formed was filtered and washed with pentane (2 x 2 mL). CH_3CN (2 mL) and Et_3N (0.04 mL, 0.32 mmol, 1.5 equiv.) were then added and the solvent was evaporated at 60 °C (10-100 mbar) for 20 min to obtain the purified amine (14 mg, 0.08 mmol, 40% yield).

^{1}H NMR (300 MHz, $CDCl_3$): δ (ppm) 7.22-7.14 (m, 4H), 6.59 (dd, *J* = 3; 6 Hz, 2H), 4.55 (br, 4H)

^{13}C NMR (75 MHz, $CDCl_3$): δ (ppm) 144.61, 137.16, 126.35, 119.94, 117.28, 111.72

Data in accordance with literature.

Reference : https://sdbs.db.aist.go.jp/sdbs/cgi-bin/landingpage?sdbsno=6655

^{1}H NMR of the purified amine:

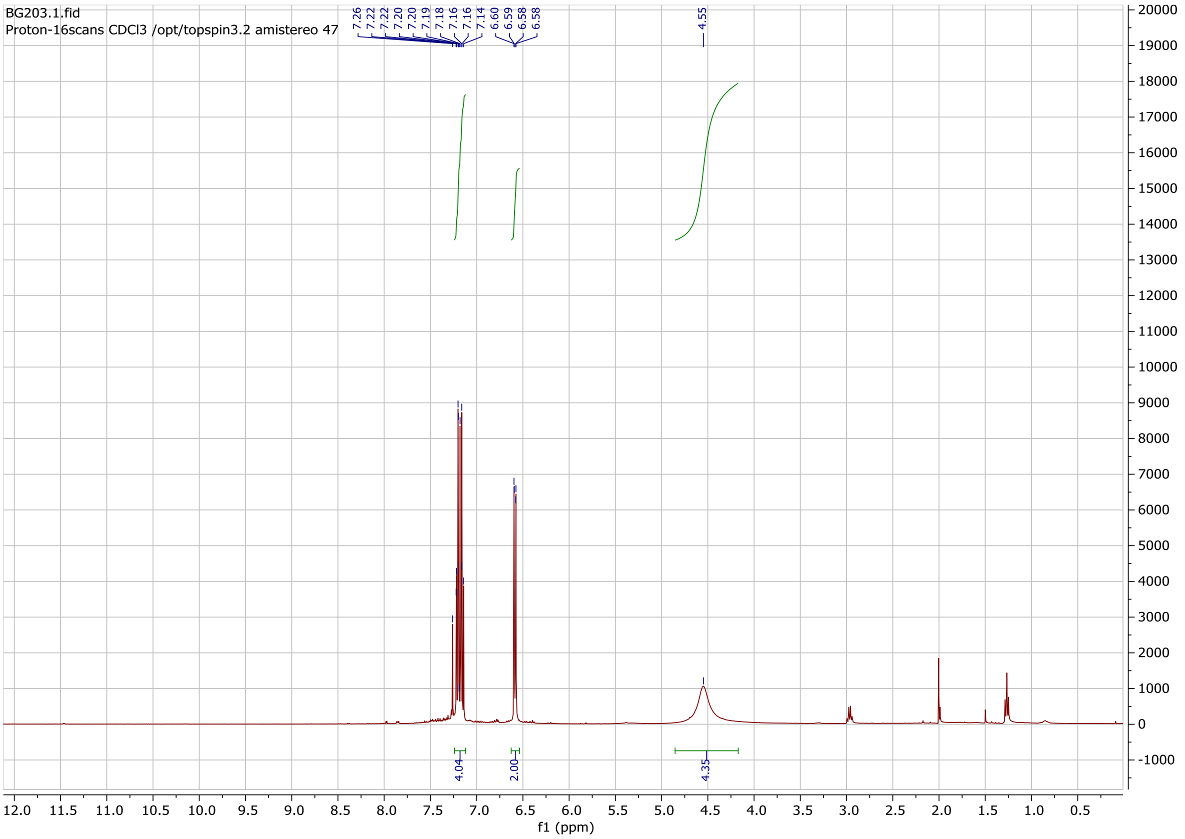

^{13}C NMR of the purified amine:

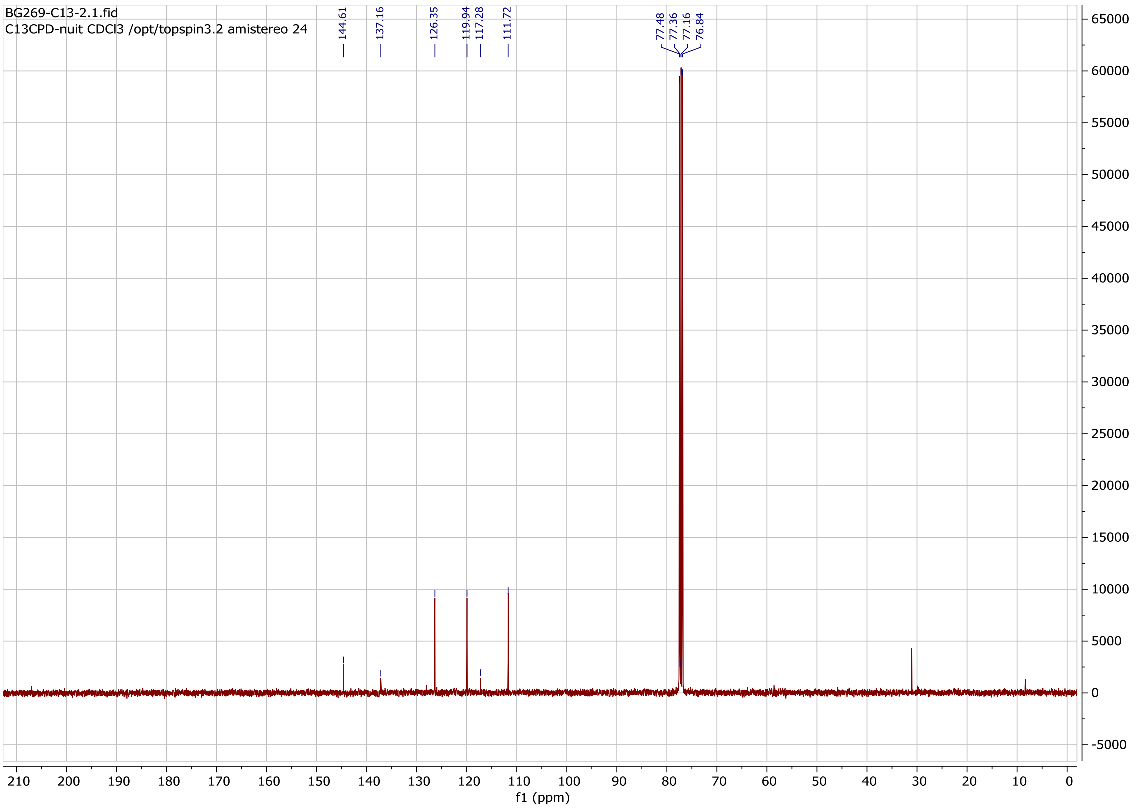

3-bromophenethylamine **5**

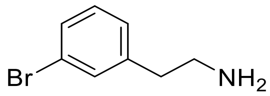

3-bromophenethylamine (42 mg, 0.21 mmol, 1 equiv.) and naphthalene (27 mg, 0.21 mmol, 1 equiv.) were dissolved in EtOAc (1 mL). TCA (100 mg, 0.62 mmol, 3 equiv.) was added at room temperature. The precipitate formed was filtered and washed with pentane (2 x 2 mL). CH_3CN (2 mL) and Et_3N (0.04 mL, 0.32 mmol, 1.5 equiv.) were added and the solvent was evaporated at 60 °C (10-100 mbar) for 15 min to obtain the purified amine (38 mg, 0.15 mmol, 71% yield).

^{1}H NMR (400 MHz, $CDCl_3$): δ (ppm) 7.34–7.32 (m, 2H), 7.17–7.1 (m, 2H), 2.95 (t, J = 6.8 Hz, 2H), 2.71 (t, J = 6.8 Hz, 2H)

^{13}C NMR (75 MHz, $CDCl_3$): δ (ppm) 142.3, 131.95, 130.13, 129.44, 127.62, 122.65, 43.34, 39.71

^{1}H NMR of the purified amine:

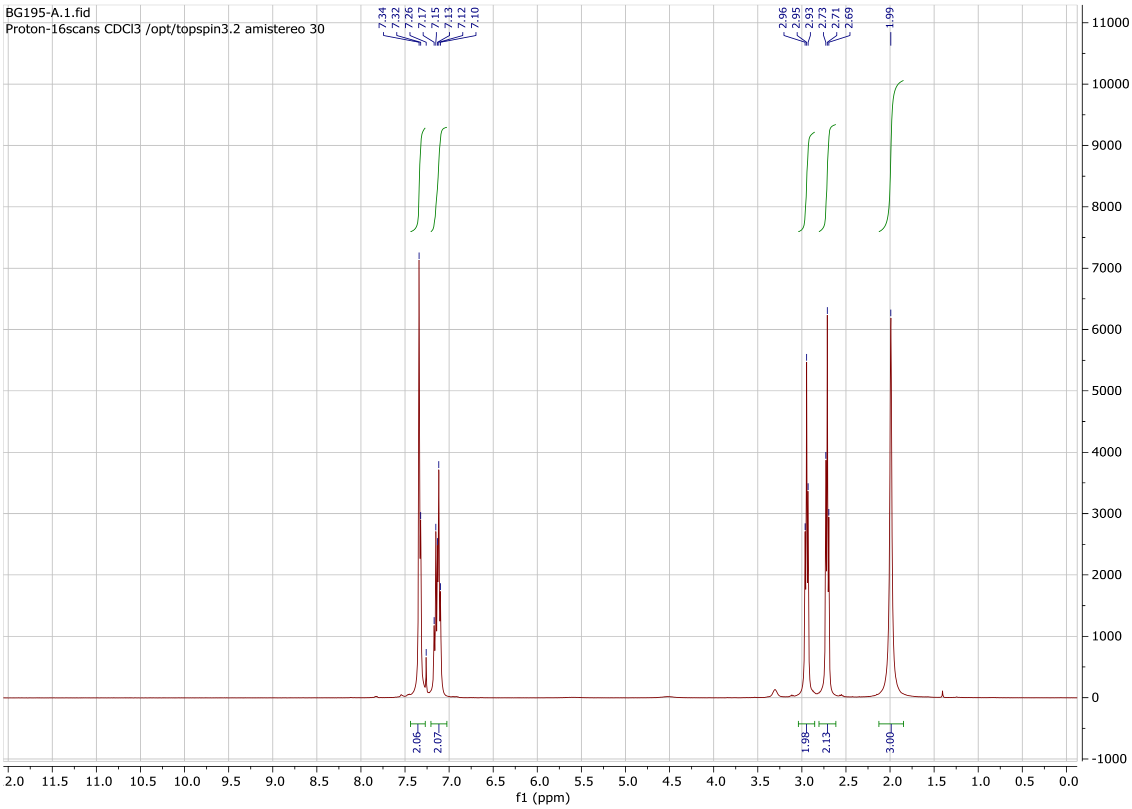

^{13}C NMR of the purified amine:

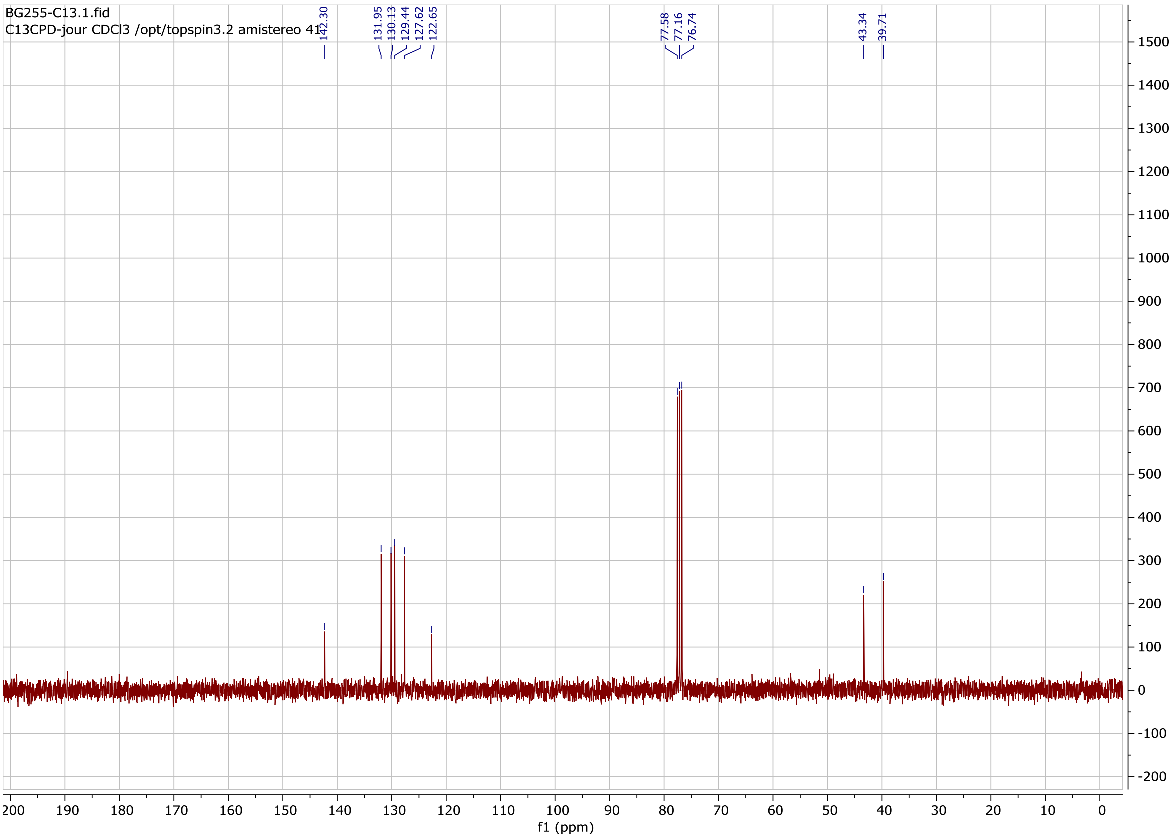

m-toluidine **6**

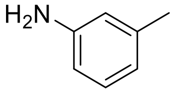

m-toluidine (24 mg, 0.21 mmol, 1 equiv.) and naphthalene (27 mg, 0.21 mmol, 1 equiv.) were dissolved in EtOAc (1 mL). TCA (100 mg, 0.62 mmol, 3 equiv.) was added at room temperature. The precipitate formed was filtered and washed with pentane (2 x 2 mL). CH_3CN (2 mL) and Et_3N (0.04 mL, 0.32 mmol, 1.5 equiv.) were added and the solvent was evaporated at 60 °C (30-100 mbar) for 15 min to obtain the purified amine (19 mg, 0.14 mmol, 79% yield).

^{1}H NMR (400 MHz, $CDCl_3$): δ (ppm) 7.05 (t, *J* = 8.0 Hz, 1H), 6.6 (d, *J* = 7.6 Hz, 1H), 6.53-6.49 (m, 2H), 3.59 (br, 2H), 2.28 (s, 3H)

^{13}C NMR (75 MHz, $CDCl_3$): δ (ppm) 146.45, 139.22, 129.26, 119.56, 116.03, 112.36, 21.52

Data in accordance with literature.

Reference : https://sdbs.db.aist.go.jp/sdbs/cgi-bin/landingpage?sdbsno=1156

^{1}H NMR of the purified amine:

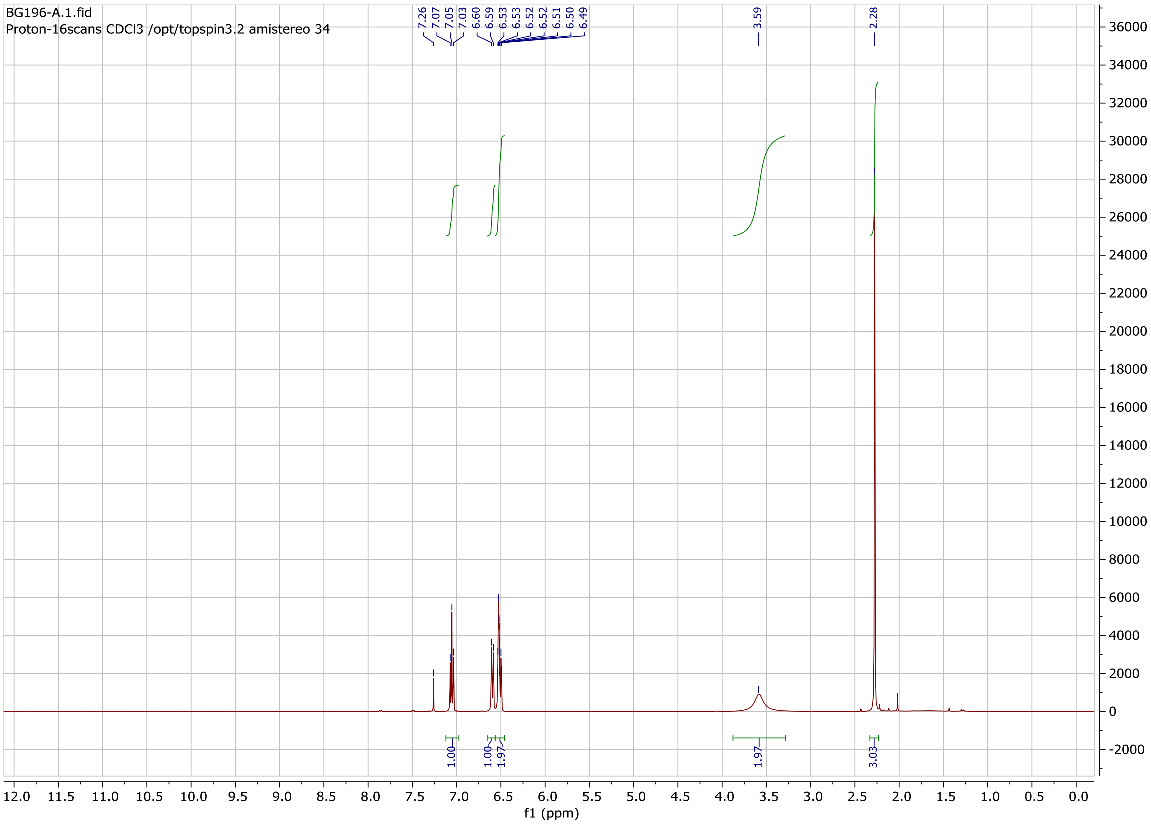

^{13}C NMR of the purified amine:

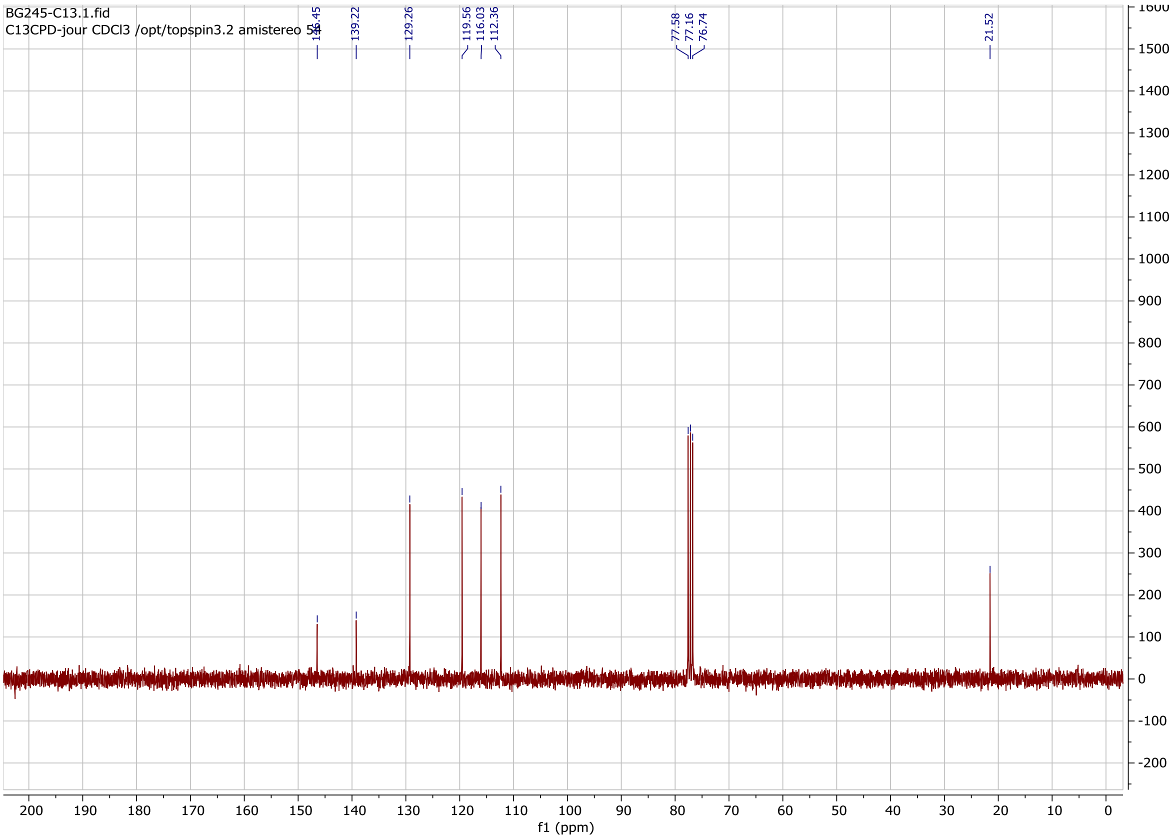

p-toluidine **7**

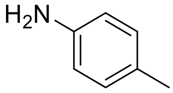

p-toluidine (28.9 mg, 0.21 mmol, 1 equiv.) and naphthalene (27 mg, 0.21 mmol, 1 equiv.) were dissolved in pentane (1 mL). TCA (100 mg, 0.62 mmol, 3 equiv.) was added at room temperature. The precipitate formed was filtered and washed with ice cold pentane (2 x 2 mL). CH_3CN (2 mL) and Et_3N (0.04 mL, 0.32 mmol, 1.5 equiv.) were added and the solvent was evaporated at 60 °C (30-100 mbar) for 15 min to obtain the purified amine (13 mg, 0.1 mmol, 45% yield).

^{1}H NMR (400 MHz, $CDCl_3$): δ (ppm) 6.98 (d, J = 8.0 Hz, 2H), 6.62 (d, J = 8.0 Hz, 2H), 3.52 (br, 2H), 2.25 (s, 3H)

^{13}C NMR (75 MHz, $CDCl_3$): δ (ppm) 143.94, 129.86, 127.89, 115.38, 20.55

Data in accordance with literature.

Reference : https://sdbs.db.aist.go.jp/sdbs/cgi-bin/landingpage?sdbsno=990

^{1}H NMR of the purified amine:

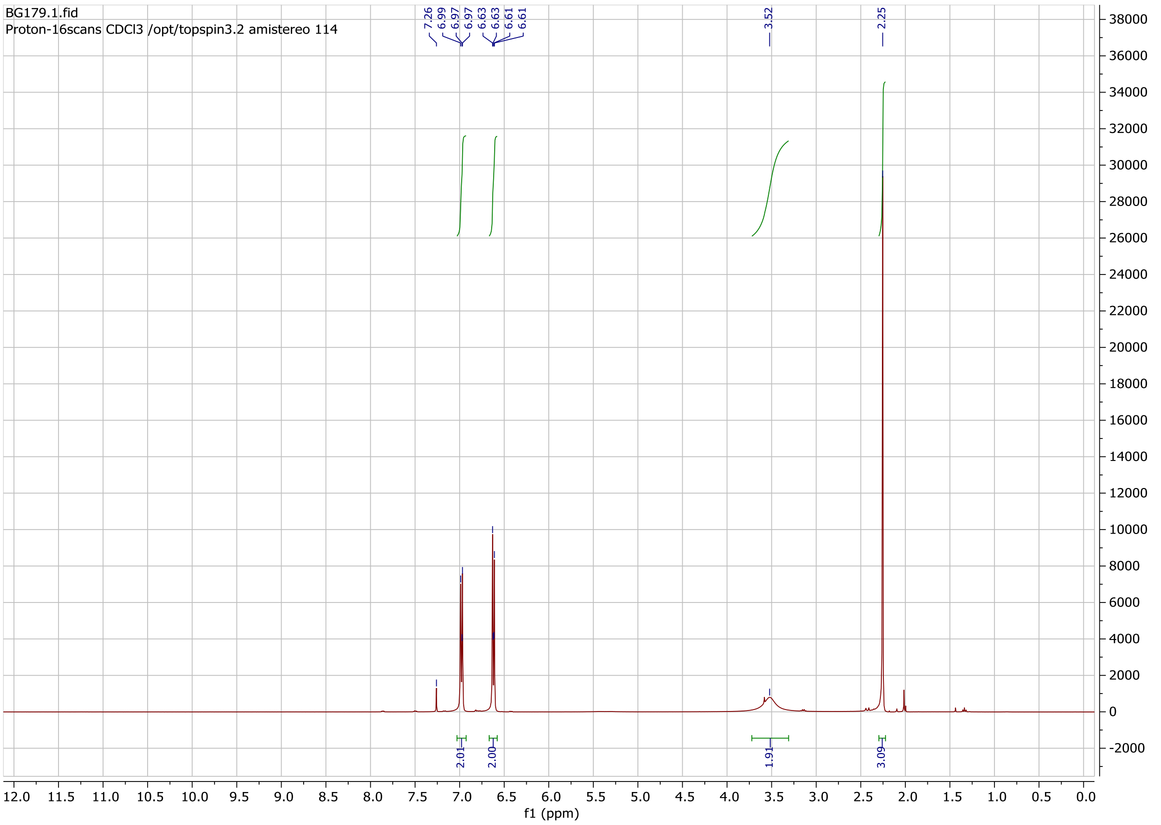

^{13}C NMR of the purified amine:

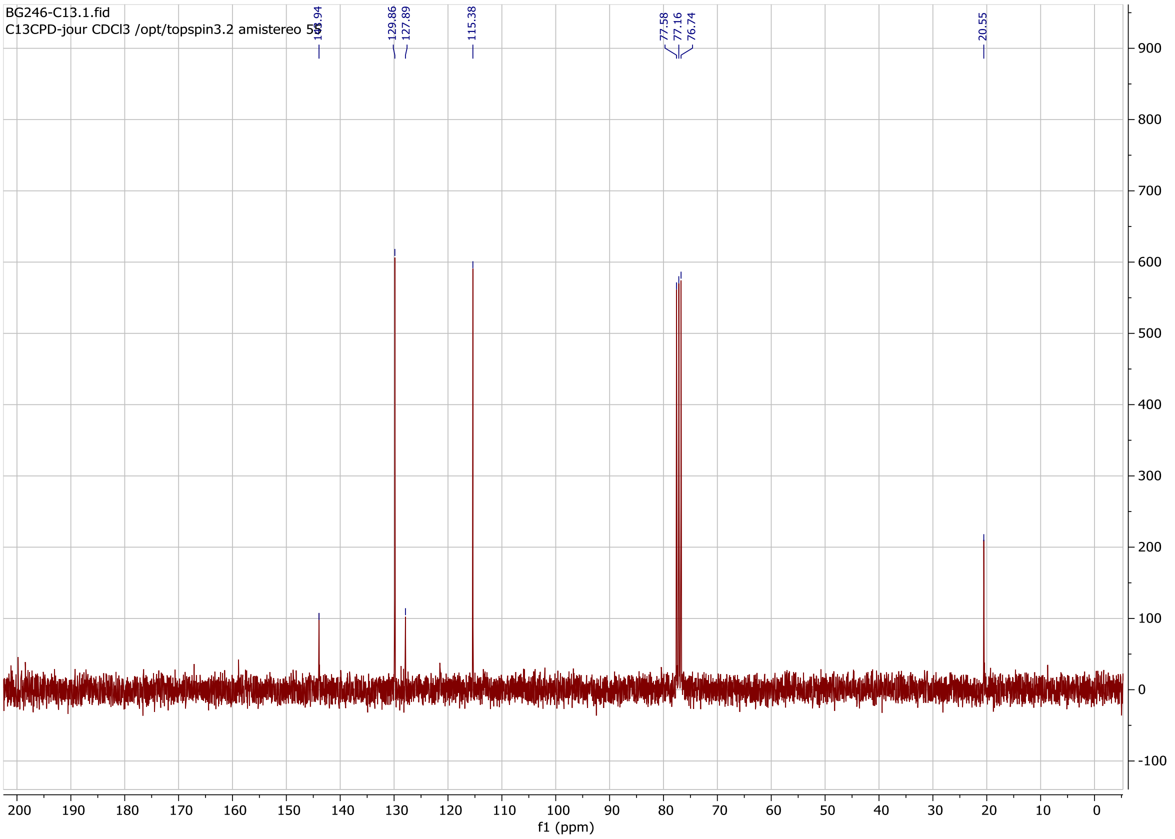

2-*t*-butylaniline **8**

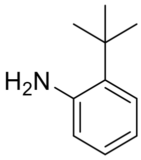

2-*t*-butylaniline (35.7 mg, 0.21 mmol, 1 equiv.) and naphthalene (27 mg, 0.21 mmol, 1 equiv.) were dissolved in pentane (1 mL). TCA (100 mg, 0.62 mmol, 3 equiv.) was added at room temperature. The precipitate formed was filtered and washed with ice cold pentane (2 x 2 mL). CH_3CN (2 mL) and Et_3N (0.04 mL, 0.32 mmol, 1.5 equiv.) were added and the solvent was evaporated at 60 °C (10-100 mbar) for 15 min to obtain the purified amine (29 mg, 0.18 mmol, 81% yield).

^{1}H NMR (400 MHz, $CDCl_3$): δ (ppm) 7.24 (d, J = 7.6 Hz, 1H), 7.04 (t, J = 7.6 Hz, 1H), 6.75 (t, J = 7.6 Hz, 1H), 6.65 (d, J = 8 Hz, 1H), 3.8 (br, 2H), 1.43 (s, 9H)

^{13}C NMR (75 MHz, $CDCl_3$): δ (ppm) 144.71, 133.83, 127.07, 126.64, 118.76, 117.89, 34.37, 29.71

Data in accordance with literature.

Reference :https://www.sigmaaldrich.com/deepweb/assets/sigmaaldrich/quality/spectra/326/001/FNMR008031.pdf

^{1}H NMR of the purified amine:

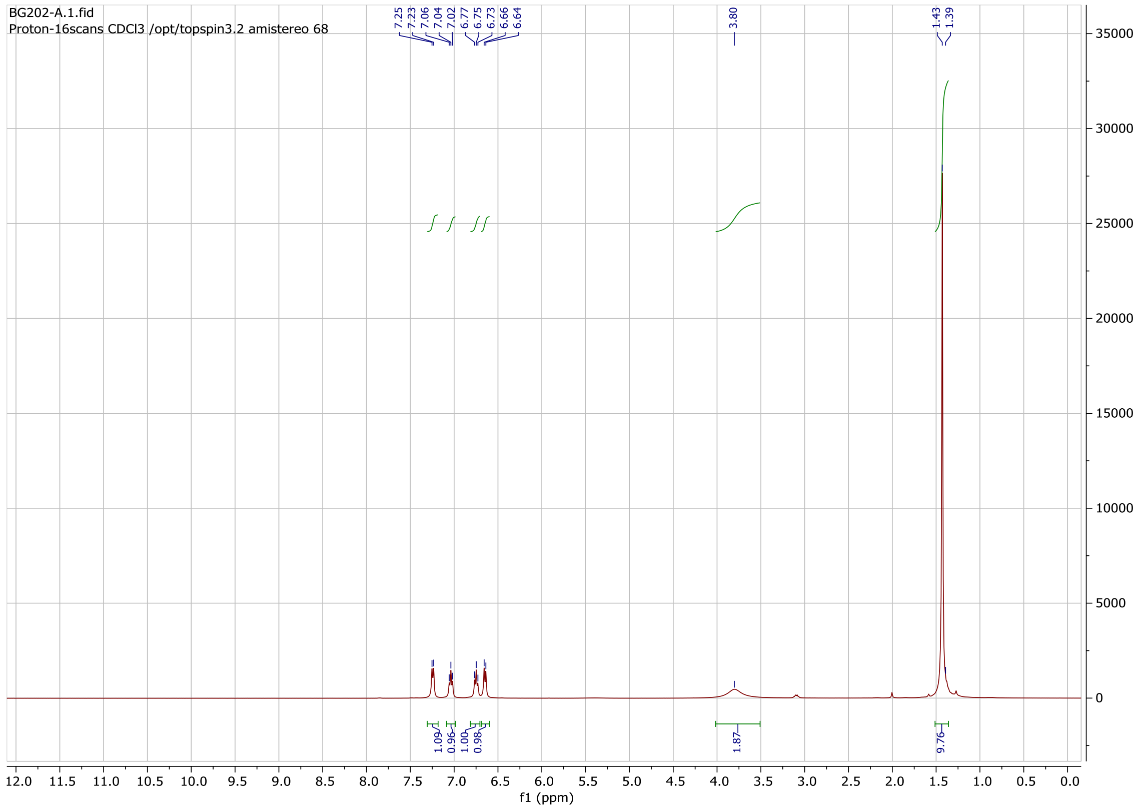

^{13}C NMR of the purified amine:

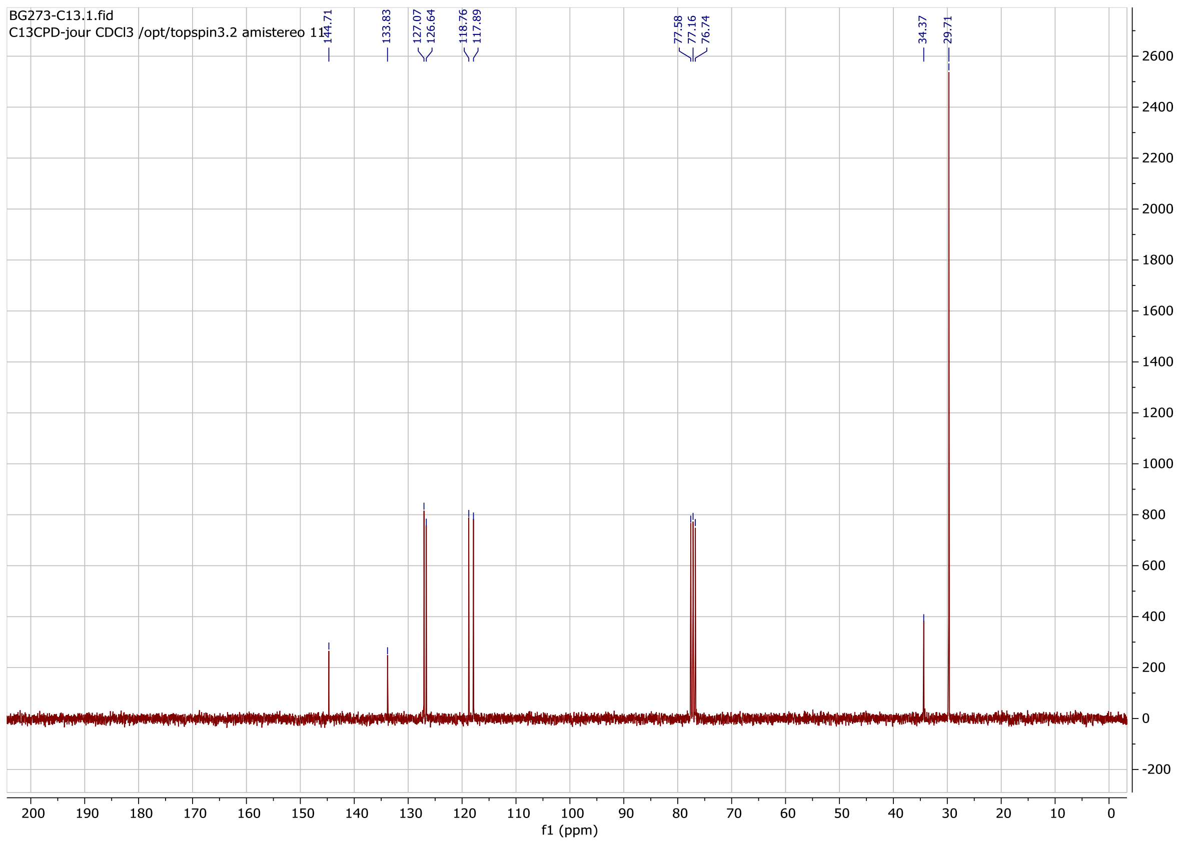

p-phenelyenediamine **9**

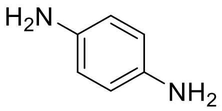

p-phenelyenediamine (23 mg, 0.21 mmol, 1 equiv.) and naphthalene (27 mg, 0.21 mmol, 1 equiv.) were dissolved in EtOAc (1 mL). TCA (100 mg, 0.62 mmol, 3 equiv.) was added at room temperature. The precipitate formed was filtered and washed with ice cold pentane (2 x 2 mL). CH_3CN (2 mL) and Et_3N (0.04 mL, 0.32 mmol, 1.5 equiv.) were added and the solvent was evaporated at 60 °C (10-100 mbar) for 15 min to obtain the purified amine (21 mg, 0.19 mmol, 91% yield).

^{1}H NMR (400 MHz, $CDCl_3$): δ (ppm) 6.57 (s, 4H) 3.27 (br, 4H)

^{13}C NMR (75 MHz, $CDCl_3$): δ (ppm) 138.73, 116.87

Data in accordance with literature.

Reference : https://sdbs.db.aist.go.jp/sdbs/cgi-bin/landingpage?sdbsno=1131

^{1}H NMR of the purified amine:

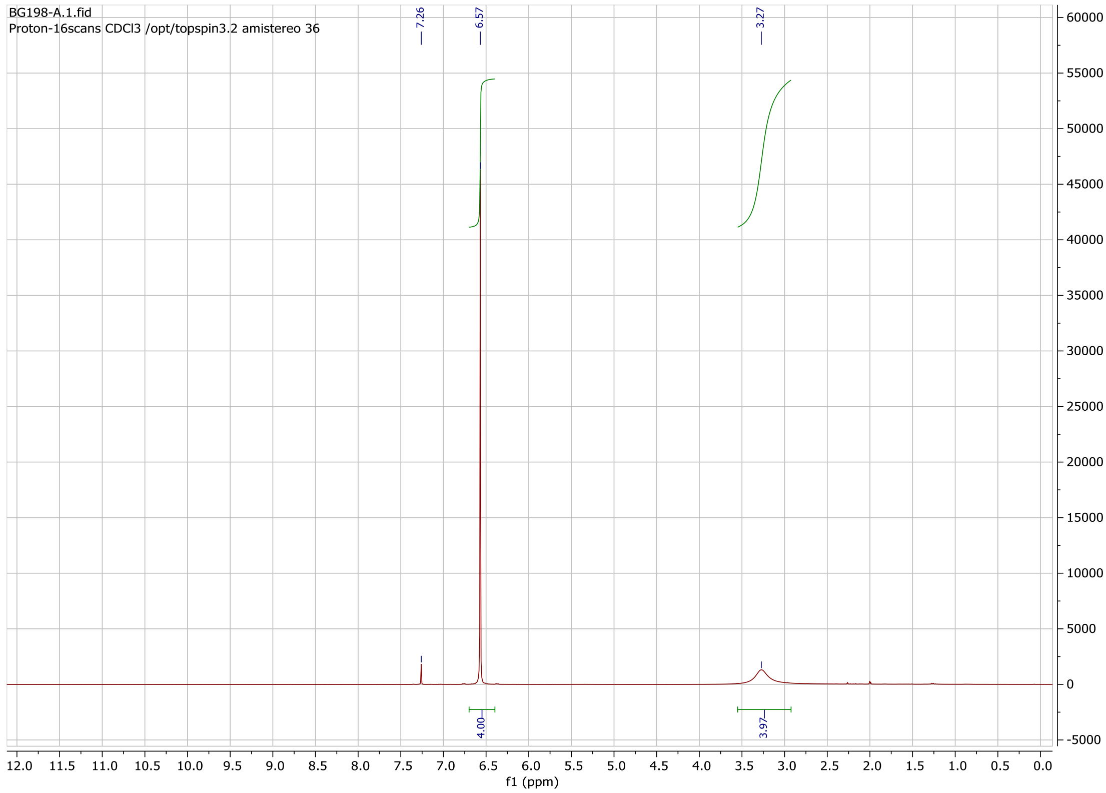

^{13}C NMR of the purified amine:

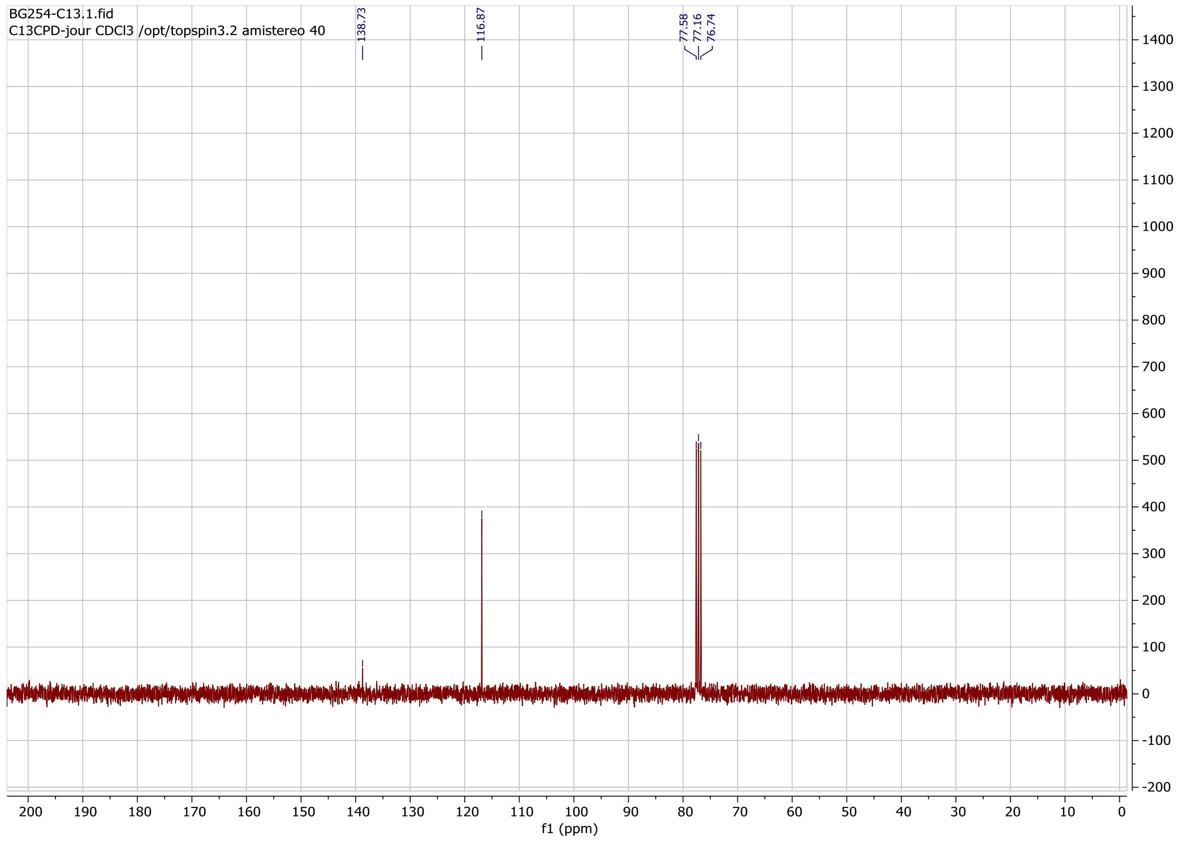

Methyl-3-amino-isonicotinate **10**

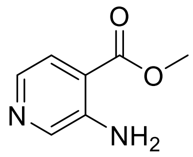

Methyl-3-amino-isonicotinate (32 mg, 0.21 mmol, 1 equiv.) and naphthalene (27 mg, 0.21 mmol, 1 equiv.) were dissolved in EtOAc (1 mL). TCA (100 mg, 0.62 mmol, 3 equiv.) was then added at room temperature. The precipitate formed was filtered and washed with pentane (2 x 2 mL). CH_3CN (2 mL) and Et_3N (0.04 mL, 0.32 mmol, 1.5 equiv.) were then added and the solvent was evaporated at 60 °C (30- 100 mbar) for 15 min to obtain purified amine (23 mg, 0.15 mmol, 72% yield).

^{1}H NMR (400 MHz, $CDCl_3$): δ (ppm) 8.18 (s, 1H), 7.9 (d, J = 4.92 Hz, 1H), 7.56 (d, J = 4.88 Hz, 1H), 5.68 (br, 2H), 3.89 (s, 1H)

^{13}C NMR (75 MHz, $CDCl_3$): δ (ppm) 167.56, 144.74, 140.84, 137.33, 122.93, 115.58, 52.16

Data in accordance with literature.

Reference : https://www.tcichemicals.com/IN/en/p/M2653

^{1}H NMR of the purified amine:

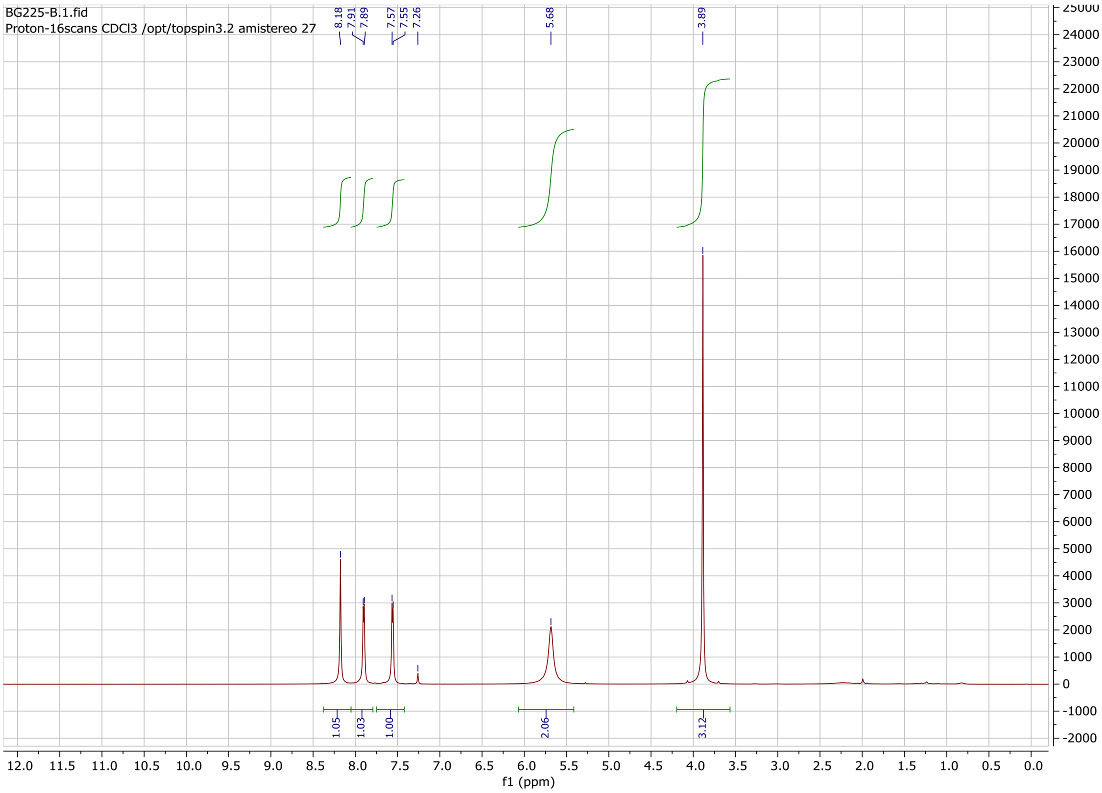

^{13}C NMR of the purified amine:

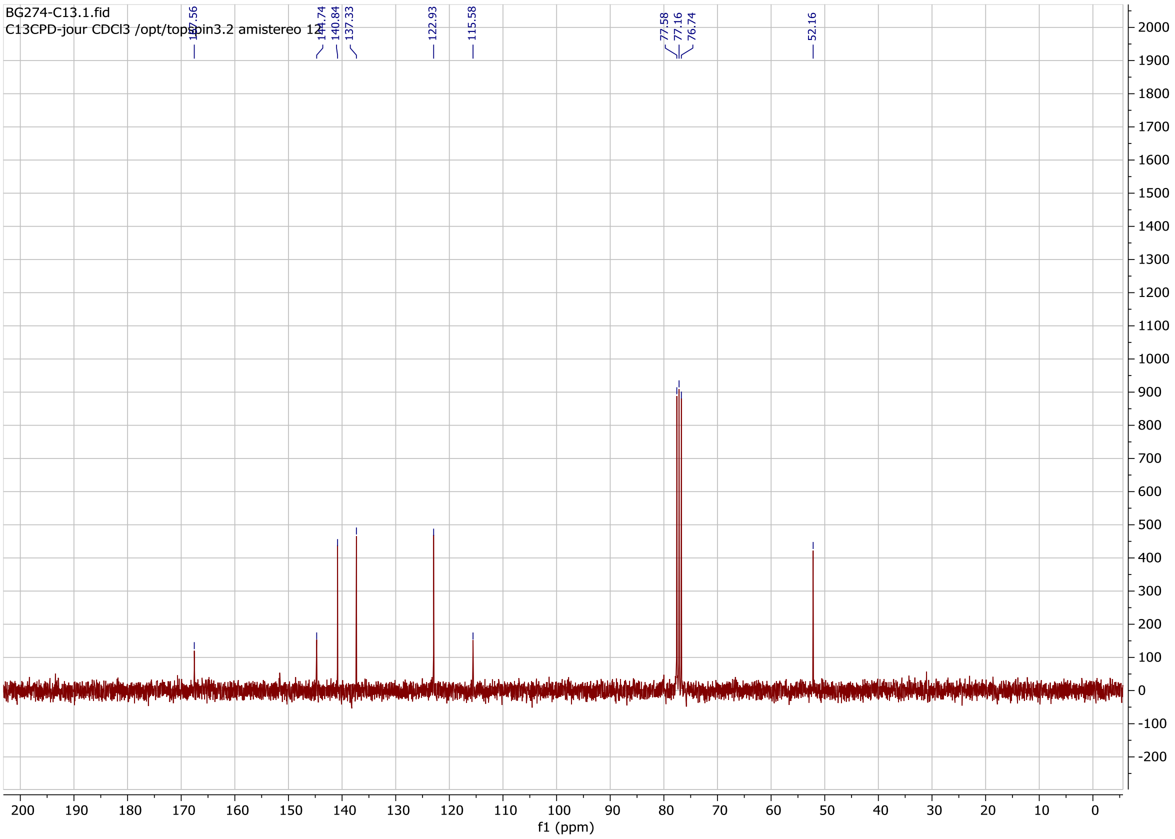

2,2,6,6-tetramethyl-4-piperidone **11**

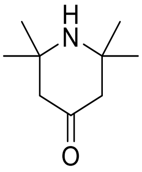

2,2,6,6-tetramethyl-4-piperidone (35.1 mg, 0.21 mmol, 1 equiv.) and naphthalene (27 mg, 0.21 mmol, 1 equiv.) were dissolved in EtOAc (1 mL). TCA (100 mg, 0.62 mmol, 3 equiv.) was then added at room temperature. The precipitate formed was filtered and washed with EtOAc (2 x 2 mL). CH_3CN (2 mL) and Et_3N (0.04 mL, 0.32 mmol, 1.5 equiv.) were then added and the solvent was evaporated at 60 °C (30- 100 mbar) for 15 min to obtain purified amine (33 mg, 0.19 mmol, 94% yield).

^{1}H NMR (400 MHz, $CDCl_3$): δ (ppm) 2.67 (s, 4H), 1.51 (s, 12H)

^{13}C NMR (75 MHz, $CDCl_3$): δ (ppm) 210.98, 55.40, 54.16, 32.06

Data in accordance with literature.

Reference : https://sdbs.db.aist.go.jp/sdbs/cgi-bin/landingpage?sdbsno=21557

^{1}H NMR of the purified amine:

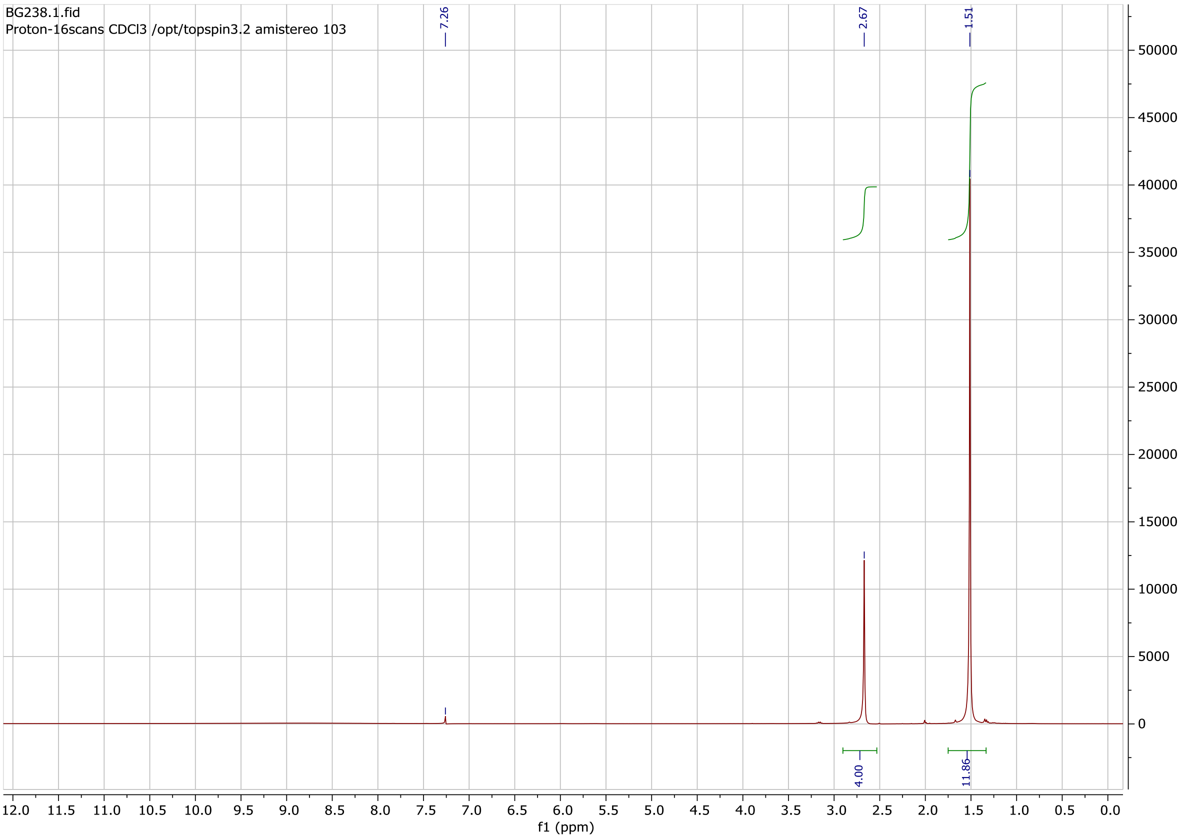

^{13}C NMR of the purified amine:

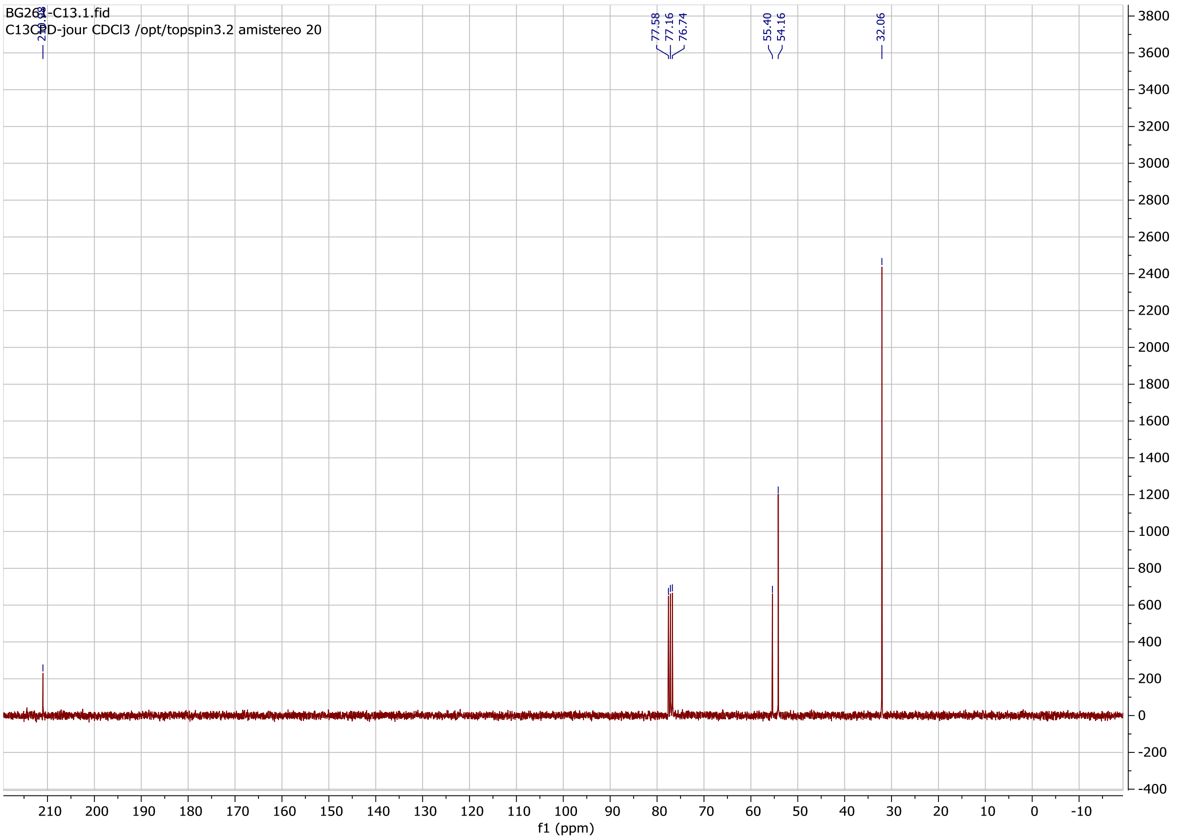

Acridine **12**

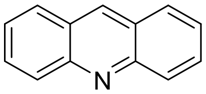

Acridine (40.2 mg, 0.21 mmol, 1 equiv.) and 2-methoxynaphthalene (33 mg, 0.21 mmol, 1 equiv.) were dissolved in Pentane-Et_2O (1 mL). TCA (100 mg, 0.62 mmol, 3 equiv.) was then added at room temperature. The precipitate formed was filtered and washed with pentane (2 x 2 mL). CH_3CN (2 mL) and Et_3N (0.04 mL, 0.32 mmol, 1.5 equiv.) were added and the solvent was evaporated at 60 °C (10-100 mbar) for 25 min to obtain the purified amine (26 mg, 0.2 mmol, 65% yield).

^{1}H NMR (400 MHz, $CDCl_3$): δ (ppm) 8.72 (s, 1H), 8.24 (d, *J* = 12 Hz, 2H), 7.97 (d, *J* = 8 Hz, 2H), 7.76 (t, *J* = 8 Hz, 2H), 7.51 (t, *J* = 8 Hz, 2H)

^{13}C NMR (75 MHz, $CDCl_3$): δ (ppm) 149.13, 136.07, 130.34, 129.46, 128.26, 126.63, 125.71

Data in accordance with literature.

Reference : https://sdbs.db.aist.go.jp/sdbs/cgi-bin/landingpage?sdbsno=1360

^{1}H NMR of the purified amine:

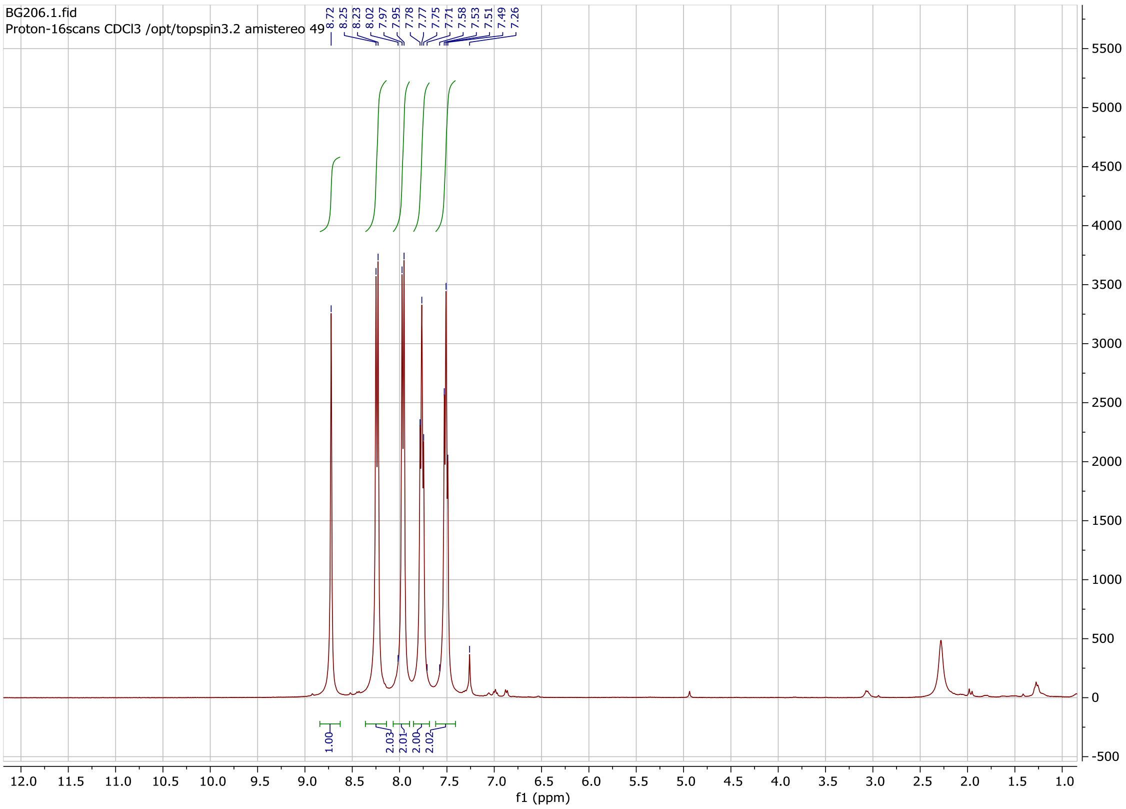

^{13}C NMR of the purified amine:

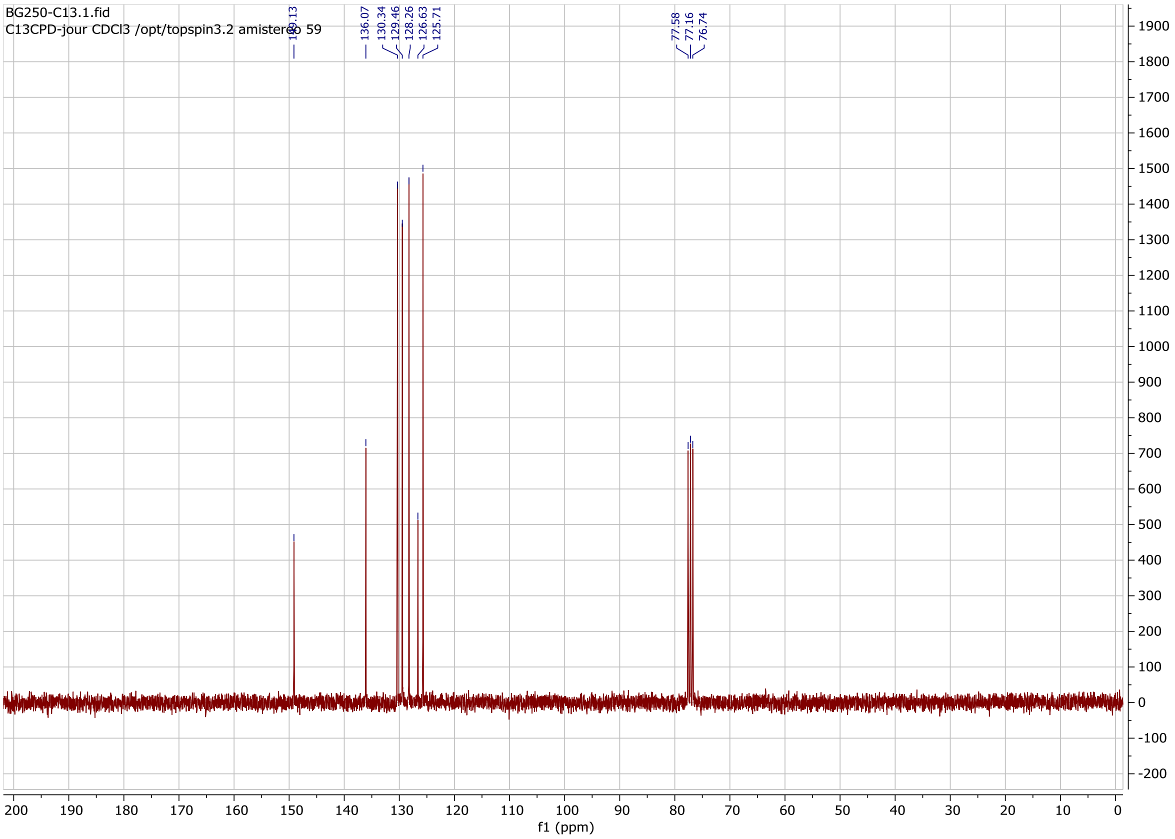

1,2-dimethylimidazole **13**

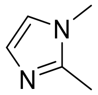

1,2-dimethylimidazole (20 mg, 0.21 mmol, 1 equiv.) and naphthalene (27 mg, 0.21 mmol, 1 equiv.) were dissolved in pentane (1 mL). TCA (100 mg, 0.62 mmol, 3 equiv.) was added at room temperature. The precipitate formed was filtered and washed with ice cold pentane (2 x 2 mL). CH_3CN (2 mL) and Et_3N (0.04 mL, 0.32 mmol, 1.5 equiv.) were added and the solvent was evaporated at 60 °C (10-100 mbar) for 15 min to obtain the purified amine (11 mg, 0.11 mmol, 53% yield).

^{1}H NMR (400 MHz, $CDCl_3$): δ (ppm) 6.87 (s, 1H), 6.76 (s, 1H), 3.54 (s, 3H), 2.35 (s, 3H)

^{13}C NMR (75 MHz, $CDCl_3$): δ (ppm) 144.99, 126.87, 120.39, 32.78, 12.83

Data in accordance with literature.

Reference : https://sdbs.db.aist.go.jp/sdbs/cgi-bin/landingpage?sdbsno=4181

^{1}H NMR of the purified amine:

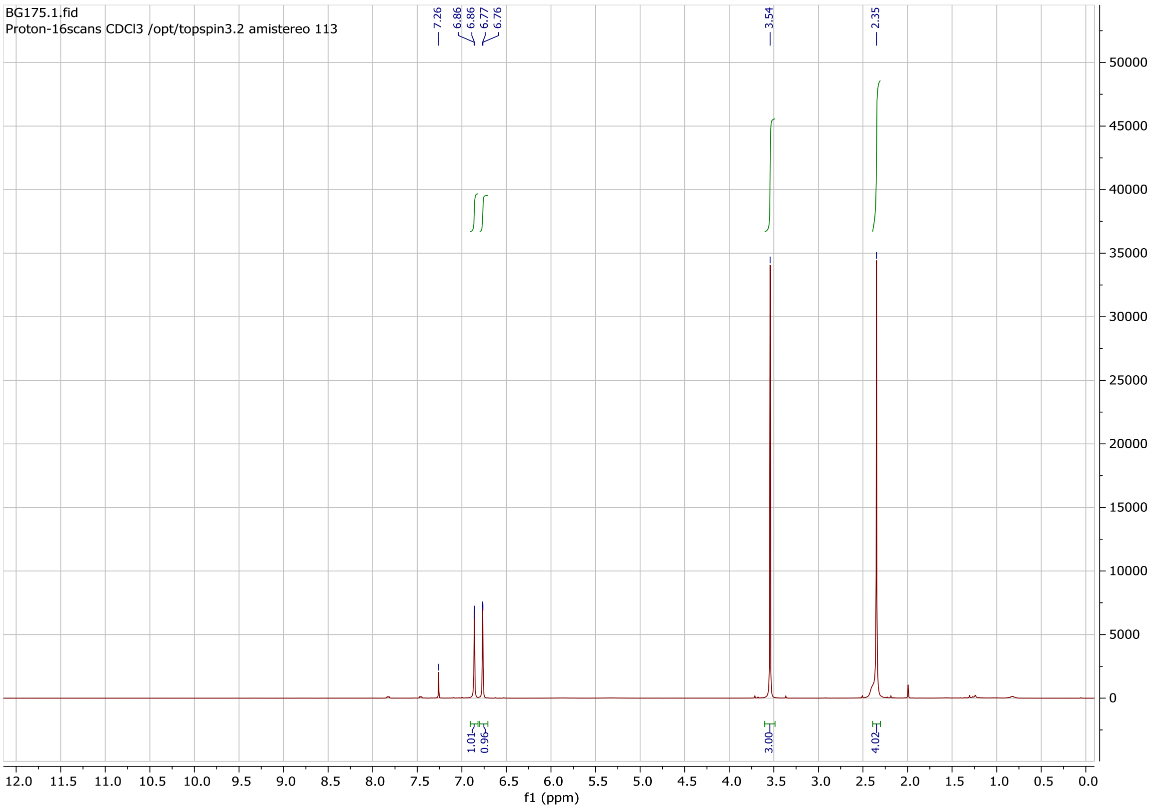

^{13}C NMR of the purified amine:

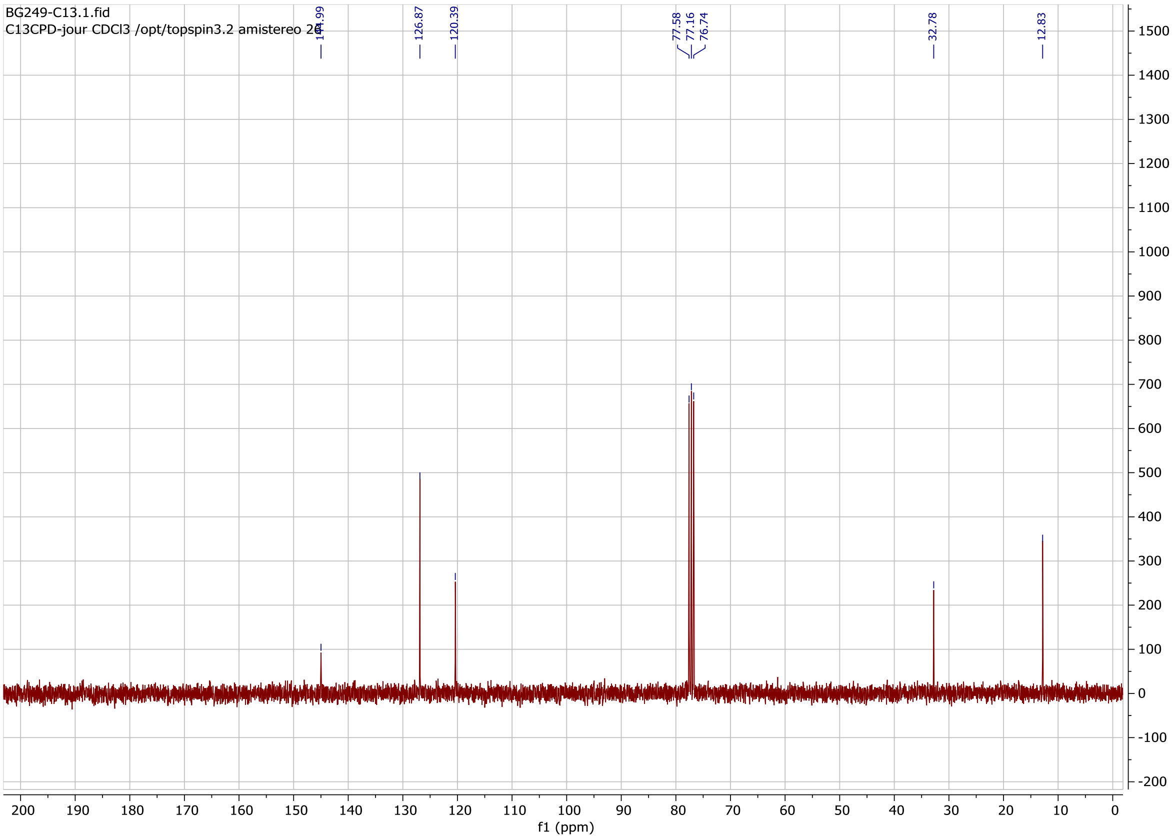

Brucin **14**

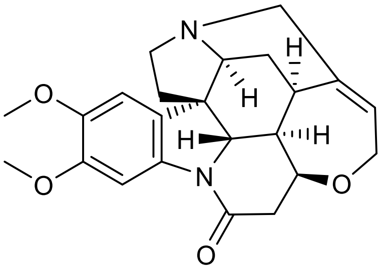

Brucin (83 mg, 0.21 mmol, 1 equiv.) and naphthalene (27 mg, 0.21 mmol, 1 equiv.) were dissolved in CH_3CN (1 mL). TCA (100 mg, 0.62 mmol, 3 equiv.) was added at room temperature. The precipitate formed was filtered and washed with ice cold pentane (2 x 2 mL). CH_3CN (2 mL) and Et_3N (0.04 mL, 0.32 mmol, 1.5 equiv.) were added and the solvent was evaporated at 60 °C (10-100 mbar) for 15 min to obtain the purified amine (49 mg, 0.12 mmol, 57% yield).

^{1}H NMR (400 MHz, $CDCl_3$): δ (ppm) 7.77 (s, 1H), 6.63 (s, 1H), 5.85 (s, 1H), 4.26-4.22 (m, 1H), 4.12-3.99 (m, 2H), 3.86 (s, 3H), 3.81 (s, 3H), 3.77 (s, 1H), 3.64 (d, J = 12 Hz, 1H), 3.14-3.02 (m, 3H), 2.83-2.76 (m, 1H), 2.68-2.58 (m, 2H), 2.41 (s, 1H), 2.33-2.27 (m, 1H), 1.88-1.77 (m, 2H), 1.42 (d, J = 12 Hz, 1H), 1.23-1.19 (m, 1H)

^{13}C NMR (75 MHz, $CDCl_3$): δ (ppm) 169.07, 149.41, 146.39, 140.59, 136.14, 127.51, 123.58, 105.78, 101.19, 77.93, 64.73, 60.51, 60.09, 56.62, 56.34, 52.83, 52.08, 50.30, 48.42, 42.57, 42.52, 31.71, 26.95

Data in accordance with literature.

Reference : https://sdbs.db.aist.go.jp/sdbs/cgi-bin/landingpage?sdbsno=4394

^{1}H NMR of the purified amine:

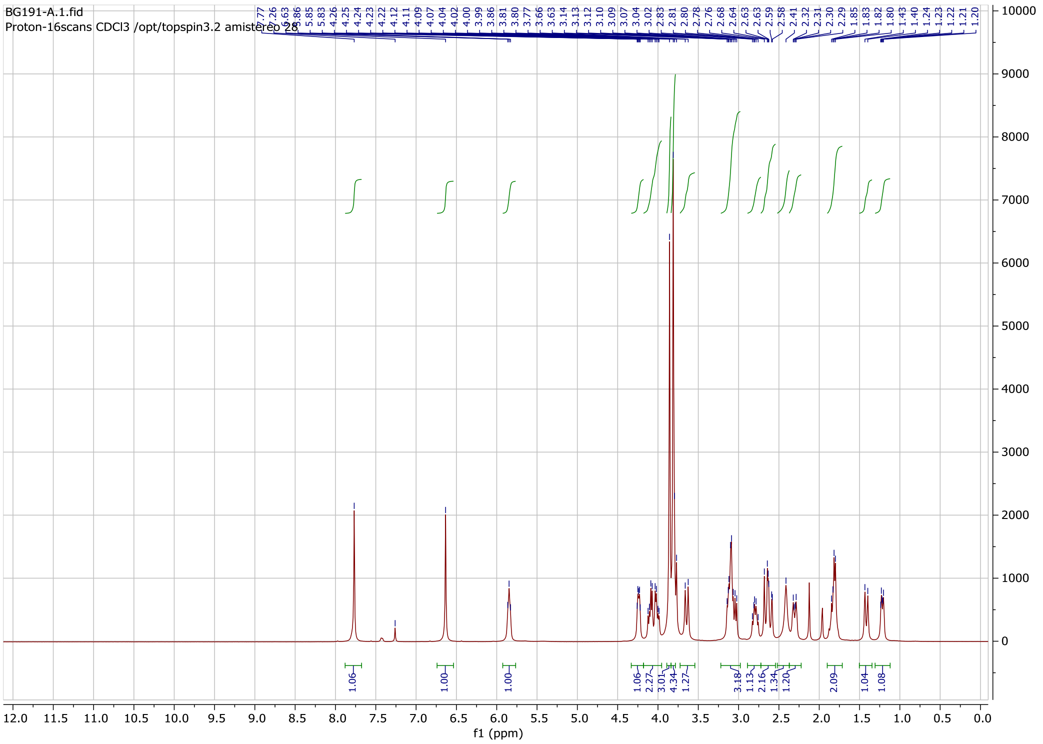

^{13}C NMR of the purified amine:

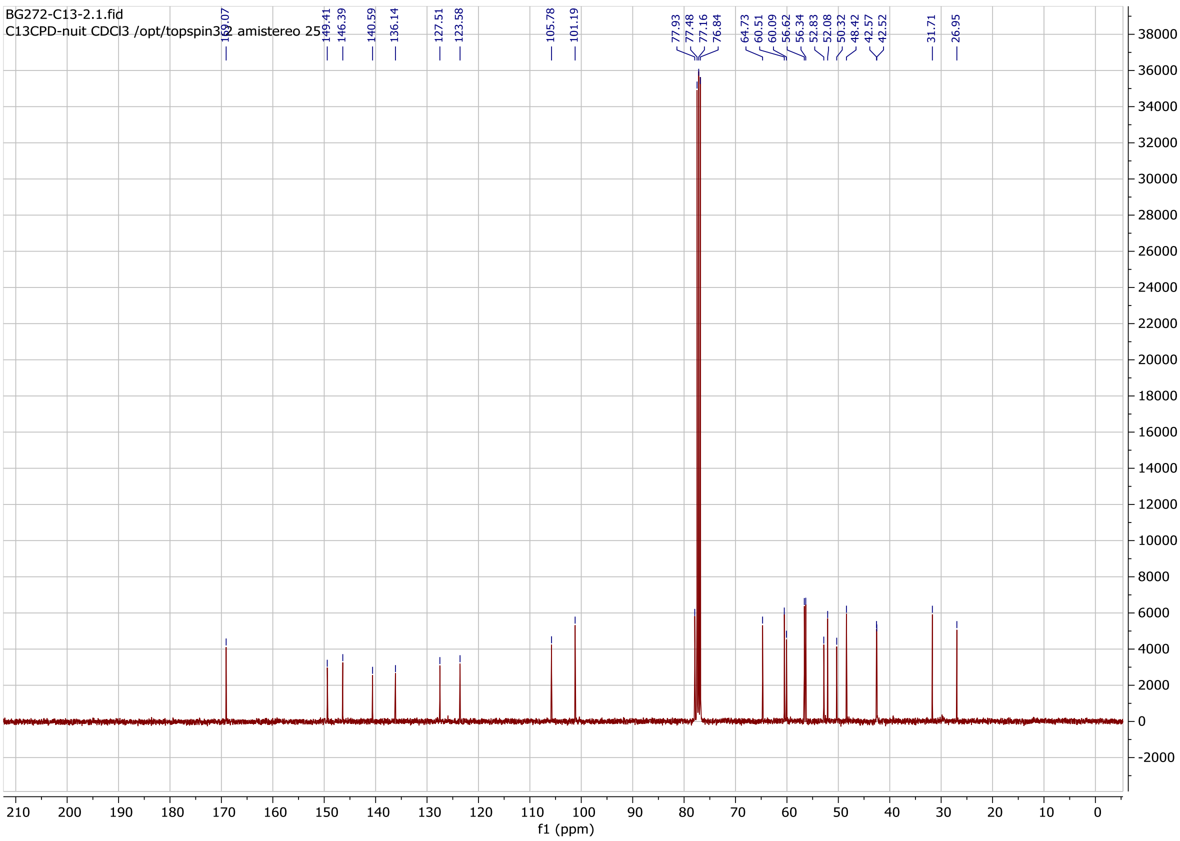

Synthesis of *p*-toluidine **7**

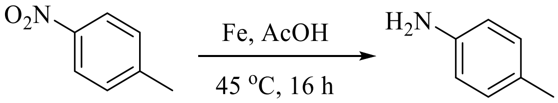

To a dry flask was added 1-methyl-4-nitrobenzene (137.14 mg, 1 mmol, 1 equiv.) and Fe (184 mg, 3.3 mmol, 3.3 equiv.). Then AcOH (5 mL) was added, and the reaction mixture was stirred at 45 °C for 16 h. After confirming the completion of the reaction by TLC, the crude reaction mixture was filtered to remove solid impurities, and the filtrate was concentrated to remove most AcOH. TCA (500 mg, 3 mmol, 3 equiv.) was then added at room temperature. The precipitate formed was filtered and washed with pentane (2 x 3 mL). CH_3CN (2 mL) and Et_3N (0.2 mL, 1.5 mmol, 1.5 equiv.) were added and the solvent was evaporated at 60 °C (30-100 mbar) for 15 min to obtain the purified amine (71 mg, 0.66 mmol, 66% yield).

^{1}H NMR (400 MHz, $CDCl_3$): δ (ppm) 6.98 (d, *J* = 7.6 Hz, 2H), 6.61 (d, *J* = 7.9 Hz, 2H), 3.36 (br, 2H), 2.26 (s, 3H)

^{13}C NMR (75 MHz, $CDCl_3$): δ (ppm) 143.81, 129.76, 127.83, 115.32, 20.45

Data in accordance with literature.

Reference : https://sdbs.db.aist.go.jp/sdbs/cgi-bin/landingpage?sdbsno=990

^{1}H NMR of the purified amine:

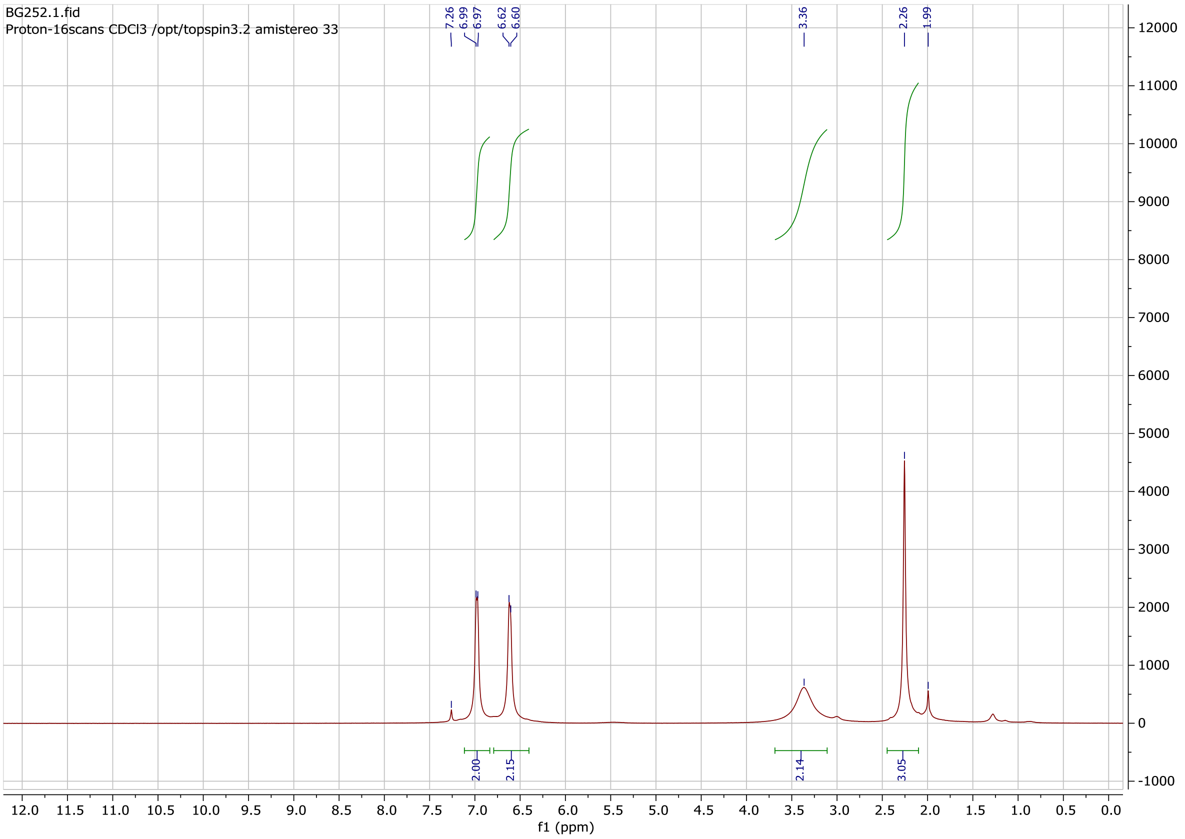

^{13}C NMR of the purified amine:

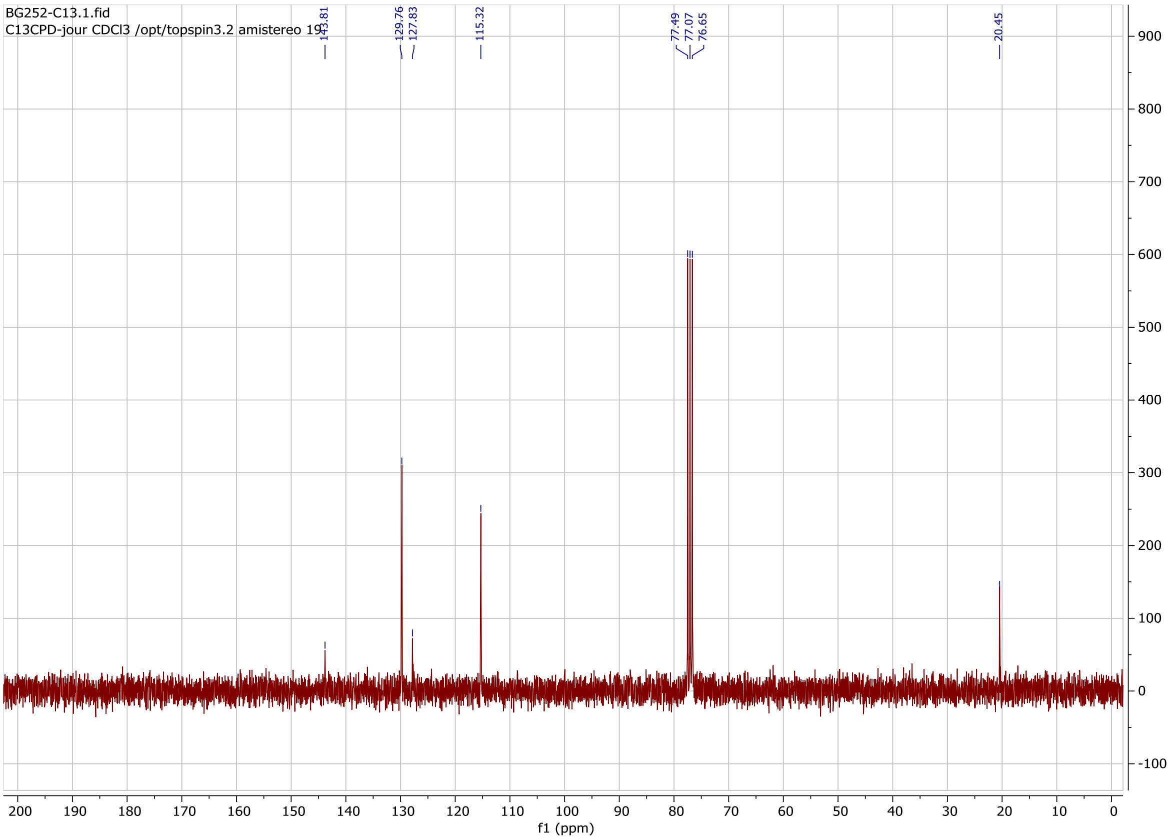

Examples of amines who did not generate a TCA salt precipitate in our first attempts:

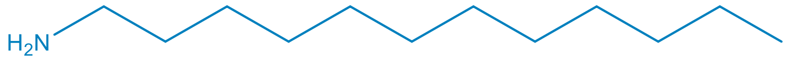

in CH_3CN, EtOAc, hexane, Et_2O

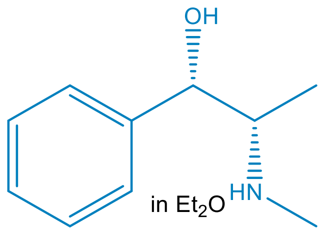

in Et_2O

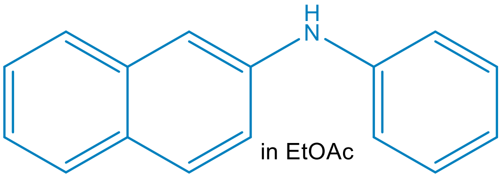

in EtOAc

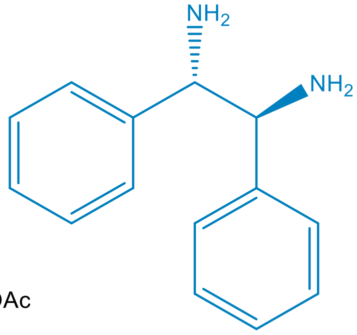

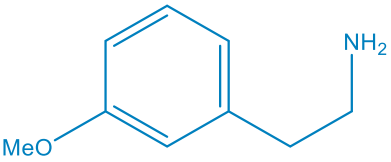

in EtOAc

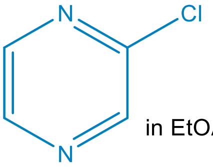

in EtOAc

in CH_3CN, EtOAc
